# MiR-137-derived polygenic risk: effects on cognitive performance in patients with schizophrenia and controls

**DOI:** 10.1038/tp.2016.286

**Published:** 2017-01-24

**Authors:** D Cosgrove, D Harold, O Mothersill, R Anney, M J Hill, N J Bray, G Blokland, T Petryshen, The Wellcome Trust Case Control Consortium, Peter Donnelly, Lesley Bates, Ines Barroso, Jenefer M Blackwell, Elvira Bramon, Matthew A Brown, Juan P Casas, Aiden Corvin, Panos Deloukas, Audrey Duncanson, Janusz Jankowski, Hugh S Markus, Christopher G Mathew, Colin N A Palmer, Robert Plomin, Anna Rautanen, Stephen J Sawcer, Richard C Trembath, Ananth C Viswanathan, Nicholas W Wood, Chris C A Spencer, Gavin Band, Céline Bellenguez, Colin Freeman, Garrett Hellenthal, Eleni Giannoulatou, Lucinda Hopkins, Matti Pirinen, Richard Pearson, Amy Strange, Zhan Su, Damjan Vukcevic, Cordelia Langford, Sarah E Hunt, Sarah Edkins, Rhian Gwilliam, Hannah Blackburn, Suzannah J Bumpstead, Serge Dronov, Matthew Gillman, Emma Gray, Naomi Hammond, Alagurevathi Jayakumar, Owen T McCann, Jennifer Liddle, Simon C Potter, Radhi Ravindrarajah, Michelle Ricketts, Matthew Waller, PaulWeston Weston, SaraWidaa Widaa, Pamela Whittaker, A Richards, K Mantripragada, M Owen, M C O'Donovan, M Gill, A Corvin, D W Morris, G Donohoe

**Affiliations:** 1 The Cognitive Genetics & Cognitive Therapy Group, The School of Psychology and Discipline of Biochemistry, The Centre for Neuroimaging & Cognitive Genomics, National University of Ireland, Galway, Ireland; 2 Neuropsychiatric Genetics Research Group, Department of Psychiatry, Institute of Molecular Medicine, Trinity College Dublin, Dublin, Ireland; 3 Institute of Psychological Medicine and Clinical Neurosciences, Cardiff University, Cardiff, UK; 4 Psychiatric and Neurodevelopmental Genetics Unit, Department of Psychiatry and Center for Human Genetic Research, Massachusetts General Hospital, Boston, MA, USA; 5 Department of Psychiatry, Harvard Medical School, Boston, MA, USA; 6 Stanley Center for Psychiatric Research, Broad Institute of MIT and Harvard, Cambridge, MA, USA; 7 MRC Centre for Neuropsychiatric Genetics and Genomics, Cardiff University, Cardiff, UK

## Abstract

Variants at microRNA-137 *(MIR137)*, one of the most strongly associated schizophrenia risk loci identified to date, have been associated with poorer cognitive performance. As microRNA-137 is known to regulate the expression of ~1900 other genes, including several that are independently associated with schizophrenia, we tested whether this gene set was also associated with variation in cognitive performance. Our analysis was based on an empirically derived list of genes whose expression was altered by manipulation of *MIR137* expression. This list was cross-referenced with genome-wide schizophrenia association data to construct individual polygenic scores. We then tested, in a sample of 808 patients and 192 controls, whether these risk scores were associated with altered performance on cognitive functions known to be affected in schizophrenia. A subgroup of healthy participants also underwent functional imaging during memory (*n*=108) and face processing tasks (*n*=83). Increased polygenic risk within the empirically derived miR-137 regulated gene score was associated with significantly lower performance on intelligence quotient, working memory and episodic memory. These effects were observed most clearly at a polygenic threshold of *P*=0.05, although significant results were observed at all three thresholds analyzed. This association was found independently for the gene set as a whole, excluding the schizophrenia-associated *MIR137* SNP itself. Analysis of the spatial working memory fMRI task further suggested that increased risk score (thresholded at *P*=10^−5^) was significantly associated with increased activation of the right inferior occipital gyrus. In conclusion, these data are consistent with emerging evidence that *MIR137* associated risk for schizophrenia may relate to its broader downstream genetic effects.

## Introduction

Genome-wide association studies (GWAS) have identified significant associations between schizophrenia (SZ) and multiple single nucleotide polymorphisms (SNPs) located within or near to the microRNA-137 (*MIR137*) host gene on chromosome 1. In the most recent and largest SZ GWAS^1^ the SNP rs1702294, located intronically to *MIR137,*^2^ was identified as the second highest SZ-associated variant. This SNP is in high linkage disequilibrium (LD) with *MIR137* variants previously identified in smaller GWAS^3^ such as rs1622579 (*r*^2^=0.99)^4^ and rs1198588 (*r*^2^=0.8).^4, 5^ In line with the proposed influence of microRNA-137 (miR-137) on proliferation, migration and maturation of neural cells,^6, 7, 8^ mechanisms important to the cognitive process, one of three intronic variants in high LD identified in this region (rs1625579) has been associated with a number of cognitively relevant phenotypes. These include lower performance in verbal episodic memory and vigilant attention,^9^ altered fronto-amygdala connectivity during a face processing task^10^ and decreased white matter integrity,^11, 12^ although a number of other studies have reported conflicting findings, with no effects of the *MIR137* risk allele^13, 14^ on brain structure.

SZ GWAS to date suggest that the disorder is likely to be highly polygenic, involving a combination of both common risk variants of small effects, such as *MIR137*, and rare variants of larger effects. Modeling or quantifying this polygenic risk is therefore important, both for the broader illness phenotypes, and for specific illness dimensions related to functional outcomes, such as cognitive deficits. It is interesting to note that miR-137 is known or predicted to regulate hundreds of other genes,^15^ many of which are independently risk loci for SZ.^3, 16^ *In vitro* studies have suggested that miR-137 targets include *BDNF*, *ZNF804A*, *TCF4*, and *CACNA1C,*^17, 18, 19, 20, 21^ variants that have been shown to impact on both cognitive performance and cortical activity by our own group and others.^13, 22, 23, 24, 25, 26^ Because miR-137’s downstream regulatory targets include loci that are independently associated with both SZ risk and with cognitive deficits, it is possible that miR-137’s regulation of these genes may also contribute to SZ-associated deficits in cognition, in addition to the *MIR137* loci’s direct effects on cognition.

The purpose of the present study was to investigate the polygenic effects on cognition of variants within genes that are downstream targets of miR-137. This was based on a previous study by Hill *et al.*,^15^ which, through miR-137 manipulation (upregulation and inhibition), assessed genome-wide transcriptional changes in a human neural progenitor cell line. This was carried out in order to elucidate pathways through which genetic disruption of miR-137 may increase SZ susceptibility. As disturbances in cognition are a cardinal feature of SZ, we investigated whether polygenic risk in this gene set explained variation in neuropsychological performance in patients and healthy participants. A key goal for this analysis was to estimate whether the variance explained by this downstream polygenic burden was independent to that explained by the single most robustly associated *MIR137* SNP, rs1702294. Our hypothesis was that a higher polygenic burden within the empirically derived miR-137 regulated gene score, excluding the rs1702294 SNP, would be associated with increased cognitive deficits in a sample of healthy participants and patients with psychosis.

## Materials and methods

### Participants

In total, 808 cases and 192 healthy participants completed a full neuropsychological assessment battery and had full genome-wide SNP data available.^1^ Cases consisted of *n*=585 clinically stable patients with a diagnosis of SZ and schizoaffective disorder (SZA), which we refer to as ‘narrow-sense’ psychosis, and an additional *n*=223 patients diagnosed with bipolar disorder with psychotic features, major depressive disorder with psychotic features, delusional disorder, or psychosis not otherwise specified who combined with SZ/SZA patients to form a ‘broad-sense’ psychosis group. Patients were diagnosed by trained psychiatrists using the Structured Clinical Interview for DSM-IV Axis I Diagnosis.^27^ Inclusion criteria required participants to be clinically stable at time of cognitive assessment, aged between 18 and 65 years, no history of co-morbid psychiatric disorder, no substance abuse in the preceding 6 months, no prior head injury with loss of consciousness, no history of seizures, and with Irish ancestry (all four grandparents born in Ireland). Healthy participants were recruited from the general population through local media advertisements. All were aged between 18 and 65 years and had Irish-born paternal and maternal grandparents, and satisfied, on the basis of clinical interview, the criteria of having no history of major mental health problems, intellectual disability or acquired brain injury, and no substance abuse in the preceding 6 months. Exclusion criteria also included having a first-degree relative with a history of psychosis. All clinical and neuropsychological assessments were conducted in accordance with the relevant ethics committees’ approval for the six sites at which this data was collected, and all participants provided written informed consent.

### Cognitive assessment

A full neuropsychological assessment designed to evaluate the cognitive deficits typically reported in SZ (general cognitive ability, memory function, attention, and social cognition) was administered to each participant. Selected subtests (Vocabulary, Similarities, Block Design, and Matrix Reasoning) of the Wechsler Adult Intelligence Scale, 3rd Edition (WAIS III)^28^ were used to measure general cognitive function. The Logical Memory (LM) I and II and Faces I and II subtests from the Wechsler Memory Scale, 3rd Edition (WMS III)^29^ were used to assess episodic and visual memory. Working memory was assessed using the Spatial Working Memory (SWM) subtest from the Cambridge Automated Neuropsychological Test Battery^30^ and Letter-Number Sequencing from the WMS III. Attentional control was assessed using the Continuous Performance Task (CPT) identical pairs version^31^ and the Sustained Attention to Response Task (SART).^32^ In addition to neuropsychological assessment, this study included measures of two aspects of social cognition—theory of mind (ToM) (frequently altered among patients with SZ and previously associated with *MIR137*^(ref. 33)^) and attributional style. ToM is the ability to attribute mental states—beliefs, intents, desires, pretending, knowledge and so on—to oneself and others and to understand that others have beliefs, desires, intentions and perspectives that are different from one's own, and is estimated using the Reading the Mind in the Eyes Task^34^ and the Hinting Task.^35^ Attributional style refers to the pervasive tendency to explain personally significant events in a particular manner.^36^ Consistency in cognitive assessment was assessed across sites by an independent researcher marking a number of patients assessed at each site.

### Genotyping

Genotyping was conducted on DNA extracted from whole blood or saliva. Full GWAS data were available for all samples. A proportion of samples were genotyped with an Affymetrix 6.0 chip (Santa Clara, CA, USA; as part of the WTCCC2,^37^ referred to as Sample A) and the remainder on the Illumina HumanCoreExome chip (San Diego, CA, USA; referred to as Sample B). SNPs were excluded on the basis of MAF<0.1%, SNP missingness ⩽2%, and Hardy–Weinberg equilibrium *P*⩽10^−6^. Imputation was carried out on these data sets separately using 1000 Genomes Phase I integrated haplotypes (Dec 2013 release) and IMPUTE2 to give ~10 million SNPs genome-wide per sample.

### Polygene score

We began by constructing the miR-137 downstream pathway based on the set of 1016 genes whose expression was identified as being altered by miR-137 manipulation in the study by Hill *et al.*,^15^ 831 of these genes could be unambiguously mapped to the autosomes and this gene set was used to generate polygene scores. We then cross-referenced with unweighted *P-*values from the PGC2 2014 GWAS^1^ for variants within 20 kb of each gene in this set without LD-pruning (scores with and without pruning were highly correlated). We next defined three arbitrary threshold values based on the PGC threshold values (*P*<10^−5^, *P*<0.05, and *P*<0.5) to generate three polygene risk score values for analysis. Using these thresholds, 1020, 20 920 and 89 102 SNPs were included in the analysis, respectively. Finally, each participant was then given a weighted polygene score based on the number of risk alleles they carried within this gene list at the given gene threshold, weighted by the log of the SZ-association odds ratio from the PGC2. Genes used in this process are found in the Supplementary Information.

### Statistical analysis: neuropsychological tests and polygene scores

Data were inspected for heteroscedasticity, and outliers,^38^ which were excluded from analysis. To estimate polygene effects on cognitive deficits associated with SZ (intelligence quotient (IQ), memory, attentional control and social cognition) linear regression analyses were performed using IBM SPSS Statistics,^39^ with age and gender entered as variables of no interest, and test score as the dependent variable. To take into account potential differences in results between Sample A and B due to the two differing genotyping platforms used, a linear regression was performed separately in each sample (those genotyped using Affymetrix and those with Illumina). Results from these were then meta-analyzed using the inverse variance method.^40^ In the inverse variance method, the weight given to each sample is chosen to be the inverse of the variance of the effect estimate (that is, one over the square of its standard error). This minimizes the imprecision of the pooled effect estimate. An estimate of sample heterogeneity and significance of such is shown in Supplementary Table 1 (*I*^2^). To maximize power to detect differences, we carried out our analysis on the full dataset of all cases and controls (*n*=1000). We then followed any significant results in the patient only groups (both the broad psychosis group (*n*=808) and narrow psychosis group (patients with SZ and schizoaffective disorder only (*n*=585)) to confirm the direction of effects in these groups (Supplementary Tables 2 and 3). As the meta-analyses showed little evidence of heterogeneity of results in Sample A and B despite the differing genotype platforms, the sample was analyzed as a whole to provide an estimate of effect size provide *r*^2^ and standardized β values (Supplementary Table 4). A *post hoc* power calculation estimated^41^ that *n*=988 has 0.88 power to detect a very conservative polygene score effect of *r*^2^=0.01, with *α*=0.05, although there are certain caveats to this type of calculation.^42^

### Functional MRI

A subgroup of the healthy participants in this study (all right handed) had also undergone functional imaging during two cognitive tasks. One hundred eight participants completed a spatial working memory task and 83 had completed a facial processing task, with tasks and acquisition parameters as described by us previously.^10, 33, 43, 44, 45^ The task can be summarized as follows.

#### Spatial working memory task

This block design task was presented using Presentation software (Neurobehavioral Systems, Albany, CA, USA). Participants were asked if a white dot was in the same spatial location as a red circle (Supplementary Figure 1) during three conditions. In the baseline condition the white dot and red circle both appear at the same time. In the 1-dot condition, the white dot and red circle are separated by a 3- s delay. Finally, in the 3-dot condition, 3 white dots and a red circle are presented, separated by a 3- s delay. Task accuracy and reaction time were also measured.

Out of 108 participants, there were 3 missing this behavioral data that were excluded from fMRI analysis, and one participant was excluded due to an error with the task that caused it to run for several seconds after the scan finished. Two additional participants were excluded from analysis due to movement and 16 were excluded from all analysis due to low-quality MRI data. This left a final sample of 86 healthy participants.

The beginning of the spatial working memory task was synchronized to the first transistor-transistor logic (TTL) pulse sent from the MRI scanner at the start of the sequence. However, for a minority of participants the start of the task was not synchronized to the TTL pulse due to an experimental error; for these individuals the task was run manually at the start of the sequence. Due to the block design nature of this task, any small discrepancies in timing resulting from this error are unlikely to affect results.

#### Face processing task

Developed by Grosbras *et al.*,^46^ participants (*n*=83) watched a series of 2-s to 5-s black and white videos of faces, which started from a neutral expression, and then turned into an angry expression or neutral expression, interspersed with a control condition consisting of video clips of black and white concentric circles expanding and contracting. Blocks of video clips lasted 18- s, with 4–7 video clips presented per block. Overall, there were 19 blocks: 5 blocks of neutral face videos, 5 blocks of angry face videos and 9 control condition blocks (every second block was a control block). To make sure that individuals had paid attention to the videos, they performed a face recognition test after scanning. In this test, 5 stil-color pictures of faces were presented and participants were asked to determine whether these matched faces seen during the task. Six participants were excluded as they scored less than 4/5 correct answers or were missing data for the face recognition task. One face processing participant was excluded from analysis due to excessive movement and 5 participants were excluded due to low-quality MRI data and/or significant artefacts, resulting in a final sample of 70 participants.

#### Imaging pre-processing and statistical analysis

Spatial pre-processing and statistical analysis of MRI data was performed using Statistical Parametric Mapping (SPM8, revision 4290, http://www.fil.ion.ucl.ac.uk/spm/software/spm8/) and MATLAB R2011b (v7.13; http://www.mathworks.co.uk/). Functional images were realigned to the mean functional image, normalized to MNI (Montreal Neurological Institute) space with a voxel size of 3 × 3 × 3 mm^3^ and smoothed using a 10 mm FWHM (full-width at half maximum) isotropic Gaussian filter. After spatial pre-processing, graphical plots of the estimated time series of translations and rotations were inspected for excessive motion, which we defined as more than 3 mm translation and/or 3° rotation.

For the spatial working memory task, statistical analysis was performed using a general linear model (GLM) with two contrasts: spatial working memory (1 dot and 3 dots versus baseline), and increased spatial working memory load (3 dots>1 dot). For the face processing task, three task conditions (angry faces, neutral faces and baseline) and four contrasts consistent with our examination of neural activity associated with this task in SZ patients:^33^ Neutral faces versus baseline, angry faces versus baseline, all faces (angry and neutral) versus baseline, and angry faces versus neutral faces.

Participants’ contrast maps were entered into a second-level analysis to investigate effects of *MIR137* network on neural activity (multiple regression analysis with the empirically derived miR-137 regulated gene score as covariate of interest). This multiple regression analysis was performed for the empirically derived *MIR137* gene score at the *P*=10^−5^, *P*=0.05 and *P*=0.5 levels. Results were examined at a *P*<0.001 (uncorrected) level and clusters were considered statistically significant at a *P*<0.05 level, family-wise error (FWE) corrected for multiple comparisons across the whole brain at the cluster level. For each of these clusters, MNI coordinates of significant maxima were entered into the Anatomy toolbox in SPM8 (refs 47, 48, 49) and probable anatomical regions were identified using the AllAreas_v18_MPM atlas.

## Results

### Participant demographics: neuropsychological tests

Patient demographic information appears in Table 1. In all cognitive tests, healthy participants performed better than the patient groups. An ANOVA found that the miR-137 polygene scores differed significantly between diagnosis groups at each threshold. Predictably, patients showed higher polygene scores than healthy participants. A *post hoc* Tukey test indicated that the polygene scores for each patient group (narrow and broad sense) was significantly different from the control group (*P*<0.001) but not from each other.

Poorer performance on measures of IQ, memory, and attention were each found to be associated with higher miR-137 polygene scores at all *P*-value thresholds. When participants were combined, significant findings were found across all three polygene thresholds in domains of IQ, episodic memory, visual memory and working memory (Table 2). As hypothesized, performance on these neuropsychological measures decreased as polygene score increased. The most markedly significant domain was for declarative memory functioning. Multiple declarative memory subtests (the logical memory [immediate/delayed] and faces subtests [immediate/delayed] of the WMS III) were highly significant at each score threshold, surviving a stringent Bonferroni correction for the five cognitive domains analyzed. In contrast, the analysis of the single miR-137 risk SNP rs1702294 only showed significant influence on three domains: IQ, attention, and a ToM measure (Table 3; *P*-values ranging from *P*=0.042 to *P*=0.007).

As a *post hoc* analysis, when only patients were considered (and not controls), the same general pattern of results were observed as for the full group, with the relationship between higher polygene scores and lower test accuracy remaining significant at the *P*=10^−5^ threshold, and nominally significant at the *P*=0.05 threshold. Similarly, when the effects of rs1702294 were considered in patients only, nominal effects of the risk allele on poorer general cognitive ability and memory ability remained. Finally, when only SZ/SZA patients (‘narrow sense psychosis’) were analyzed, the same direction of results was also observed for both the polygene (Supplementary Tables 2 and 3) and single SNP analysis (Table 3). The effects of polygene risk on cognitive test performance were broader than that seen for the rs1702294 SNP alone (Supplementary Table 4). This was the case across each of the whole, broad psychosis and narrow psychosis samples. Whereas the risk ‘C’ allele of rs1702294 was associated with variance on only three cognitive tests, the polygenic risk was associated with multiple test scores in both the memory and general cognitive domains.

### Participant demographics: fMRI

Participant demographics for the fMRI spatial working memory sample are presented in Table 4. A regression analysis was performed in SPSS (22.0.0) to examine any significant effects of *MIR137* pathway risk (at *P*=10^−5^, *P*=0.05 and *P*=0.5 levels) on age, gender, years of education, SWM accuracy or SWM reaction time in our sample. Overall, there were no significant effects of *MIR137* pathway risk on any of these variables (*P*>0.05).

Participant demographics for the overlapping fMRI face processing sample are presented in Supplementary Table 5. A regression analysis was performed in SPSS (22.0.0) to examine any significant effects of *MIR137* pathway risk (at *P*=10^−5^, *P*=0.05 and *P*=0.5 levels) on age, gender, or years of education, and once again there were no significant effects of *MIR137* pathway risk on any of these variables (*P*>0.05).

### Effects of MIR137 pathway risk score on neural activity

**Figure 1:**
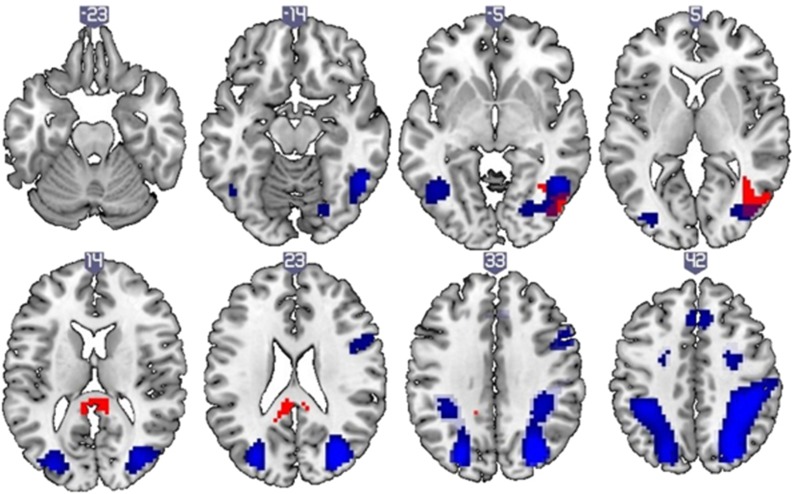
Effect of increasing *MIR137* pathway risk score (*P*=10^−5^ level) on neural activity during increasing spatial working memory load (3 dots versus 1 dot), 3 outliers removed (*N*=83; shown in red), shown alongside overall activation for this contrast in the same group (shown in blue). Cluster 1 (right inferior occipital gyrus and right middle temporal gyrus), top panels; Cluster 2 (medial parietal region) bottom panels. Clusters are significant at *P*<0.05, FWE-corrected at the cluster level and represent regions where neural activity was greater with increasing risk score. Each 2D axial slice is labeled with an x-coordinate (MNI space). Clusters are rendered on the ‘ch256’ brain template using MRIcroGL (http://www.mccauslandcenter.sc.edu/mricrogl/). Additional editing of figure (for example, changing the size/resolution) performed using MS Paint and/or Paint.NET v3.5.10. FWE, family-wise error; *MIR137*, microRNA-137; MNI, Montreal Neurological Institute.

Increasing *MIR137* pathway risk score (*P*=10^−5^ level) was associated with significantly increased neural activation in clusters incorporating the right middle temporal gyrus, left posterior cingulate and left thalamus with increasing spatial working memory load (3 dots versus 1 dot contrast, Supplementary Table 6). In each individual the mean parameters estimates of all voxels was calculated for each cluster showing a significant effect. These values were then entered into SPSS to check for outliers. Overall, three outliers were detected. As such, the analysis was run again, excluding these individuals. When this analysis was re-run, two clusters showed a significant effect of increasing *MIR137* pathway risk score, one incorporating the right inferior occipital gyrus and right middle temporal gyrus, and another in the medial parietal region (individual cluster peaks not found on any probability maps using Anatomy toolbox). We observed that, in the high working memory load condition (3-dot versus 1-dot) increasing *MIR137* polygenic risk score (*P*=10^−5^ level) was associated with increased activation across two clusters (Supplementary Table 6). The first incorporated the right inferior occipital gyrus and right middle temporal gyrus and showed peak coordinates in the right inferior occipital gyrus (MNI peak coordinates 48, −76, −2; *t*_max_=4.80; *P*-value=0.011; cluster extent (voxels)=175; Figure 1). A second cluster, located in the medial parietal region, did not localize to any known probability maps using Anatomy toolbox (Cluster extent [voxel]=121, *P*=0.038, *t*_max_=4.19; MNI peak coordinates 3, −34, 16), possibly because these coordinates represent a region of overlap between gray and white matter (Figure 1). No other effects were observed either for other *MIR137* polygenic thresholds or other contrasts. Note that neural activity associated with the 3-dot versus 1-dot condition across all participants is presented in Supplementary Table 7 and Figure 1, showing overlap with the right inferior occipital cluster in which a *MIR137* effect was observed.

Finally, for the face processing task, after correcting for outliers (*n*=1 using the same outlier detection method as for the spatial working memory data) no significant effects of the empirically derived miR-137 regulated gene score (at *P*=10^−5^, *P*=0.05, or *P*=0.5 levels) were observed for any other contrast.

## Discussion

This study assessed the effects of higher polygenic risk burden in a network of genes whose expression was altered by manipulation of miR-137 expression, the gene product of a SZ risk gene. On the basis of previous studies from our group and others, we sought to establish whether this gene network would convey the same cognitive and cortical effects as have been associated with individual risk variants at this locus and, if so, whether the amount of variance explained was the same or greater. We did this based on polygene scores derived from all risk variants within this network (not including *MIR137* risk SNPs themselves), calculated from three risk thresholds using the PGC-SCZ^1^ data (*P*=10^−5^, *P*=0.05 and *P*=0.5). Following correction for multiple testing we found that, irrespective of the threshold used, genetic variants within the defined *MIR137* score were associated with lower declarative memory performance. We also found that the *MIR137* pathway was nominally associated with poorer working memory and general cognitive ability. Finally, functional imaging revealed that participants showed increased cortical activation in right inferior occipital gyrus during a spatial working memory task. This was found in the absence of any association with cortical activity during a face processing task and polygenic risk. Collectively, these data indicate deleterious effects of risk variants within the empirically derived miR-137 regulated gene score was on cognitive function in patients and controls.

Studies seeking to characterize the cognitive effects of individual risk variants have been criticized previously for low power, making their findings difficult to replicate. This study, based on one of the largest single data sets available (*n*=1000 individuals with adequate genetic information available from a total of 1269 participants collected) attempted to overcome this difficulty through the use of a relatively large sample size and a novel approach to risk—that is, calculated on the basis of a risk gene molecular pathway. The robustness of our results are supported by the following: (1) the direction of all significant results were the same both for the full dataset and when both the narrow and broad patients groups were analyzed alone; (2) the findings were consistent across all genetic risk thresholds, even after correction for the number of cognitive functions assessed (general cognitive ability, declarative memory, working memory, attentional control, and social cognition) was made; (3) in the case of the memory subtests used, findings replicated across modalities, that is, increased polygenic risk was associated with both poorer verbal performance and poorer non-verbal performance; finally, (4) the amount of variation in cognitive performance explained by the empirically derived miR-137 regulated gene score was larger than that explained by the single *MIR137* variant, rs1702294, with the polygenic pathway score explaining in the region of ~2% (Supplementary Table 1) of variance on declarative memory measures. Although still modest, this is approximately two to three times the variance explained by the individual rs1702294 SNP.

Although correction for multiple testing was carried out for the cognitive component of the study (a Bonferroni correction for five domains: IQ, working memory, visual declarative memory, attention and social cognition) we did not correct for the fact that three polygenic risk significance thresholds were used. It is still unclear what the best approach to selecting thresholds is. The original PGC analysis^3^ reported 10 risk thresholds. We have included three to reduce the multiple testing burden, while others have reduced this burden even further by including only one, for example, a threshold of *P*=0.5.^50^ However, for our cognitive analysis the threshold considered would appear to have been unimportant: broadly the same cognitive associations were observed for each of the three thresholds considered. This would suggest that in fact a study of only one threshold would have been sufficient to characterize the cognitive consequences of this gene set. For the neuroimaging study, an association between *MIR137* polygenic risk and cortical activation during a working memory task was only significant at the most conservative threshold of *P*=10^−5^; why this might have been case is uncertain. One possibility is that this was simply a false-positive finding; the fact that only a subsample of participants were included in this analysis, albeit relatively large for fMRI studies (*n*=108) might support this conclusion. Other explanations include that imaging findings may be more sensitive to the signal to noise ratio inherent in high polygenic risk thresholds (that is, that more lenient thresholds harbor a greater percentage of variants not robustly associated with risk, hence making them less informative). To our knowledge, only one other polygenic fMRI study has been carried out to date, and as noted included only one threshold.^50^ As a consequence this relatively novel approach requires the accumulation of further studies to determine the best thresholding approach to take, as evidence for an optimum threshold (in terms of signal to noise ratio) has yet to emerge.

When examining brain regions showing increased blood oxygenation level dependent (BOLD) response with increasing spatial working memory load (contrast: 3 dots condition>1 dot condition), two clusters showed significantly increased BOLD response in individuals with higher *MIR137* pathway risk score (*MIR137* pathway risk score *P*=10^−5^ level). The first cluster incorporated the right inferior occipital gyrus and right middle temporal gyrus. Given that this cluster overlaps with cortical regions activated by our task (Figure 1), hyperactivation in this cluster may represent relative inefficiency in processing task stimuli in individuals with higher risk scores, although this suggestion remains speculative.

Although a second cluster, in the medial parietal region, showed significantly increased BOLD response with increasing *MIR137* pathway risk score (*P*=10^−5^ level) also, no individual peak coordinates in MNI space were not found on any probability map (Figure 1). Given that visual inspection of this cluster reveals that a large proportion resides in white matter, this may be an artefactual finding and should be interpreted with caution. It is possible that as regions of the medial parietal cortex typically show task-induced decreases in BOLD response,^51^ the present finding of increased BOLD response in this region may relate to a relative failure to deactivate this region during the working memory task in individuals with higher risk scores. However, as noted above, the fact that these results are obtained in a sample that, although large for imaging studies, is quite modest for genetics studies, requires caution in interpretation of these results.

In addition to the issue of sample size, further study limitations include that only healthy participants were used for studying the association of polygenic risk on patterns of brain activation. Ideally the analysis will be repeated in a patient sample to confirm findings in this group. However, symptoms in SZ and cognitive deficits occur along a spectrum, so despite the lack of patient fMRI data, our current analysis does allow for investigation of the association between this polygene score and functional alterations in healthy participant endophenotypes. In addition, a recent paper evaluating the effect of *MIR137* on gray matter concentration found that the effects of SNPs in *MIR137* and associated pathways have greater impact on brain structure and function in SZ patients than healthy participants.^52^ The results of the present analysis of healthy participants may be indicative of a greater impact in a patient population. Another potential limitation here is using a gene list derived from human progenitor neural cells, in which the stability of transcriptional regulation can change over time. Nonetheless, at least some of the genetic variants identified to generate the polygenic score are present in both adult and embryonic stages, so the effect of the variant may have already had its substantial influence in neurodevelopmental stages. With regard to the effect of medication on cognition, medication dosage was not used as a covariate in our whole sample analysis, which included controls, as doing so would have limited our sample size in the analysis (*n*=1000) to patients with this information (*n*=585). In the patient groups considered separately, results from the neuropsychological results were non-significant following Bonferroni correction; including medication dosage as a covariate in this analysis did not change these results.

Taking a polygene approach to characterize genetic effects has particular significance for the *MIR137* polygene score. As with other gene sets and putative biological pathways, discrepancies exist between genes that are bioinformatically predicted to be related, and those that have been empirically identified. For the *MIR137* polygene scores generated here, we included only empirically derived genes. Gene expression changes in this study were found to significantly correlate with those following miR-137 manipulation in another human neural progenitor cell line in the subsequent study.^21^ Several lines of evidence suggest that at least part of the risk for SZ conferred by genetic variation at the *MIR137* locus is via effects of miR-137 perturbation on the expression of other SZ susceptibility genes.^1, 15, 20, 53^ Thus, variants within this empirically derived miR-137 regulated gene score that contribute to SZ polygenic risk may be hypothesized to impact upon cognitive function that is a central feature of the disorder. This study, by charactering the cumulative polygenic effects of these downstream risk variants while excluding the effects of risk variants with the miR-137 gene itself supports this hypothesis, both at a cognitive and at a cortical level. Functional studies indicate that miR-137 is involved in proliferation, migration and maturation of neural cells,^6, 7, 8^ likely to be the mechanisms behind this involvement with the cognitive process. Further studies of the effects of *MIR137* polygene scores may benefit from adopting a more fine-grained approach to the selection of SNPs. This could include parsing the effects on cognition of those variants in this network that are directly versus indirectly influenced by *MIR137,* given that both are likely to have been included in the gene set.^15^ This was not possible in this study as the method of gene selection could not differentiate between direct and indirect interactors of *MIR137*.

In conclusion, these data suggest that increased polygenic risk in a gene set consisting of downstream targets of miR-137 was associated with decreased cognitive performance, both in terms of memory function, and for general cognitive ability. Notably, the strength of association was stronger for the gene set as a whole than for the most strongly associated *MIR137* SNP. These data are consistent with emerging evidence that at least some of the genetic risk for SZ conferred by *MIR137* variation relates to its downstream effects on gene expression.

## Figures and Tables

**Table 1:** Participant demographic and genotype information – neuropsychological test sample

*Variable*	*Whole sample*	*Broad psychosis*	*Narrow psychosis (SZ/SZA)*	*Healthy participants*
*N*	1000	808	585	192
Age, mean (s.d.)	41.40±12.74	42.88±12.46	42.07±12.54	35.20±12.04
Female sex, %	37.7	34.4	29.9	51.6
WAIS FSIQ, mean (s.d.)	99.03±22.21	92.17±19.49	90.34±18.38	121.20±14.68
CPZE, mean (s.d.)	—	445.81±467.35	512.35±496.69	—
rs1702294 genotype %, TT/TC/CC	2.8/29.0/65.7	2.5/29.9/67.5	2.8/30.3/66.9	4.2/28.9/66.8

Abbreviations: CPZE, chlorpromazine equivalent; FSIQ, full-scale IQ; IQ, intelligence quotient; SZ, schizophrenia; SZA, schizoaffective disorder; WAIS, Wechsler Adult Intelligence Scale.

Mean±s.d. reported.

**Table 2:** Inverse variance meta-analysis results at the three defined *MIR137* polygene score thresholds for all participants

*Neuropsychological variable*	P*=10^−5^*				P*=0.05*				P*=0.5*			
	*B*	*CI*			*B*	*CI*			*B*	*CI*		
	*Combined sample*	*Lower*	*Upper*	P*-value*	*Combined sample*	*Lower*	*Upper*	P*-value*	*Combined sample*	*Lower*	*Upper*	P*-value*
Pre-morbid IQ (WTAR)	−42.477	−148.624	63.671	0.433	−503.302	−1165.205	158.601	0.136	−1671.986	−5226.713	1882.740	0.357
Verbal IQ	−303.427	−584.882	−21.972	0.035*	−4618.646	−7987.354	−1249.938	0.035*	−15034.193	−30768.408	700.022	0.035*
Performance IQ	−139.662	−359.382	80.059	0.213	−3515.312	−5765.215	−1265.410	0.002**	−9699.454	−17083.868	−2315.041	0.010*
Full-scale IQ	−217.145	−434.944	0.653	0.051	−3959.537	−6193.356	−1725.718	0.001**	−11042.282	−18376.914	−3707.651	0.003**
Logical memory 1	−308.570	−474.475	−142.666	0.000***	−3641.810	−5037.080	−2246.541	0.000***	−10669.811	−11909.440	−6125.766	0.000***
Logical memory 2	−221.538	−318.807	−124.269	0.000***	−2747.838	−3757.958	−1737.718	0.000***	−8330.306	−11909.440	−4751.172	0.000***
Faces 1	−94.815	−153.364	−36.267	0.002**	−1013.981	−1626.683	−401.278	0.002**	−2492.285	−4493.001	−491.569	0.002**
Faces 2	−71.837	−129.448	−14.226	0.015*	−791.795	−1394.458	−189.133	0.015*	−1534.968	−3504.421	434.485	0.015*
SWM (errors)	200.546	−45.339	446.430	0.110	3260.415	−465.077	6985.908	0.086	9254.991	91.947	18418.036	0.048*
SWM (strategy)	39.889	−45.908	−45.908	0.362	710.783	−186.172	1607.738	0.120	994.328	−1929.699	3918.354	0.505
Letter-number sequencing	−36.605	−69.831	−3.378	0.031*	−621.413	−967.522	−275.304	0.031*	−1732.880	−2867.928	−597.833	0.031*
SART reaction time	2048.904	−255.171	4352.979	0.081	19926.956	6182.706	33671.206	0.004**	45561.862	−1929.699	3918.354	0.505
CPT d’Prime 2 digit	−9.986	−26.034	6.061	0.223	−9.181	−181.359	162.997	0.917	128.901	−430.676	688.477	0.652
CPT d’Prime 3 digit	−1.246	−16.494	14.002	0.873	19.569	−142.902	182.039	0.813	243.740	−204.455	691.934	0.286
CPT d’Prime 4 digit	5.034	−7.721	17.790	0.439	46.054	−89.501	181.609	0.505	229.518	−213.062	672.099	0.309
Eyes	27.701	−39.150	94.553	0.417	−61.410	−748.152	625.332	0.861	−96.271	−2371.226	2178.684	0.934
Hint	−23.224	−80.013	33.566	0.423	−503.302	−1165.205	158.601	0.136	−1553.490	−3541.015	434.036	0.126
Externalizing bias	18.604	−30.143	67.352	0.454	194.019	−327.109	715.147	0.466	211.583	−1473.479	1896.644	0.806
Personalizing bias	−0.546	−7.551	6.458	0.878	−23.491	−97.795	50.812	0.535	−59.643	−263.523	144.236	0.566

Abbreviations: CI, confidence interval; CPT, Continuous Performance Task; IQ, intelligence quotient; *MIR137*, microRNA-137; SART, Sustained Attention to Response Task; SWM, Spatial Working Memory; WTAR, Wechsler Test of Adult Reading.

Results in bold indicate significance after correction for multiple testing across 5 cognitive domains.

*P<0.05; **P<0.01; ***P<0.0001.

**Table 3:** Linear regression analyses of rs1702294 risk allele (C) effects on cognitive test scores for all participants

*Neuropsychological variable*	*Standardized β*	*CI*		P*-value*	r*^2^*
		*Lower*	*Upper*		
*IQ*					
Pre-morbid IQ (WTAR)	−0.044	−2.215	0.482	0.207	0.002
Verbal IQ	−0.046	−4.514	0.709	0.153	0.002
Performance IQ	−0.091	−6.701	−1.041	0.007**	0.008
Full-scale IQ	−0.080	−6.157	−0.563	0.019*	0.006
					
*DM*					
Logical memory 1	−0.050	−3.077	0.298	0.106	0.003
Logical memory 2	−0.025	−1.740	0.726	0.420	0.001
Faces 1	−0.059	−1.322	0.151	0.119	0.003
Faces 2	−0.008	−0.815	0.646	0.820	0.000
					
*WM*					
SWM (errors)	0.031	−1.611	4.415	0.361	0.001
SWM (strategy)	0.017	−0.800	1.316	0.632	0.000
Letter-number sequencing	−0.032	−0.642	0.202	0.306	0.001
					
*Attention*					
SART reaction time	0.059	−3.873	30.073	0.013*	0.003
CPT d’Prime 2 digit	−0.044	−0.302	0.103	0.334	0.002
CPT d’Prime 3 digit	−0.031	−0.257	0.126	0.504	0.000
CPT d’Prime 4 digit	−0.019	−0.194	0.127	0.679	0.000
					
*Social cognition*					
Eyes	−0.040	−1.308	0.449	0.337	0.001
Hint	−0.083	−0.972	−0.018	0.042*	0.008
Externalizing bias	0.000	−0.613	−0.614	0.998	0.000
Personalizing bias	0.020	−0.033	0.053	0.645	0.000

Abbreviations: CI, confidence interval; CPT, Continuous Performance Task; DM, declarative memory; IQ, intelligence quotient; SART, Sustained Attention to Response Task; SWM, Spatial Working Memory; WM, working memory; WTAR, Wechsler Test of Adult Reading.

Results in bold indicate significance after correction for multiple testing across 5 cognitive domains.

*P<0.05; **P<0.01; ***P<0.0001.

**Table 4:** Participant demographics: fMRI sample

Age	28.79±9.05^a^
Gender	38 M/48 F
Years of education	17.48±3.32^b^
SWM accuracy (correct trials/72)	63.59±8.77
SWM reaction time (ms)	8737.15±2166.99
*MIR137* pathway risk score at *P*=10^−5^	0.047755814±0.006577924
*MIR137* pathway risk score at *P*=0.05	0.022951163±0.000631104
*MIR137* pathway risk score at *P*=0.5	0.014223256±0.000195643

Abbreviations: *MIR137*, microRNA-137; SWM, Spatial Working Memory.

a Mean±s.d. reported.

b Education information available for 82 of 86 participants.
