# Supplementary material for: MiR-137-derived polygenic risk: effects on cognitive performance in patients with schizophrenia and controls
Source: Transl Psychiatry. 2017 Jan 24;7(1):e1012–. doi: 10.1038/tp.2016.286 (PMC5545742; doi:10.1038/tp.2016.286)
Supplement: Supplementary Information [file tp2016286x1.docx]

**Supplementary Tables 1-3: Results of Meta-Analyses for Whole Sample, Broad Psychosis and Narrow Psychosis Samples at Defined Polygene Score Thresholds (*p*=10^-5^*, p*=0.05, *p*=0.5).**

A proportion of samples were genotyped with an Affymetrix 6.0 chip (Sample A) and the remainder on the Illumina HumanCoreExome chip (Sample B). To take into account the two different genotyping platforms, a linear regression was performed separately in each sample as described in the methods section. Results shown include B (unstandardized coefficient) value estimation for each sample and a combined value based on combined samples; along with B confidence intervals and *p* values.

I^2^ describes the variability in effect estimates that is due to heterogeneity rather than sampling error (chance).

As described by Higgins and Green (2008), a rough guide to interpretation is as follows:

- 0% to 40%: might not be important;
- 30% to 60%: may represent moderate heterogeneity*;
- 50% to 90%: may represent substantial heterogeneity*;
- 75% to 100%: considerable heterogeneity*.

*The importance of the observed value of I^2^ depends on (i) magnitude and direction of effects and (ii) strength of evidence for heterogeneity (e.g. P value from the chi-squared test, or a confidence interval for I^2^).

**Supplementary Table 1.1:** Inverse variance meta-analysis results at *p*=10^-5^ *MIR137* polygene risk score threshold for whole sample

|  |  | Sample A |  | Sample B |  | Combined Sample | 95% CI | Combined Sample |  | Heterogeneity | |
| --- | --- | --- | --- | --- | --- | --- | --- | --- | --- | --- | --- |
|  | Neuropsychological Variable | (n=597) |  | (n=403) |  |  | Lower | Upper | *p* | I^2^ | *p* |
|  |  | B | r^2^ | B | r^2^ | B |  |  |  |  |  |
| IQ | Pre-morbid IQ (WTAR) | -53.125 | 0.001 | -23.846 | 0 | -42.477 | -148.624 | 63.671 | 0.433 | 0 | 0.795 |
|  | Verbal IQ | -177.168 | 0.003 | -466.754 | 0.019 | -303.427 | -584.882 | -21.972 | 0.035* | 0.447 | 0.179 |
|  | Performance IQ | -139.698 | 0.002 | -139.545 | 0.002 | -139.662 | -359.382 | 80.059 | 0.213 | 0 | 1 |
|  | Full-scale IQ | -188.348 | 0.003 | -291.097 | 0.008 | -217.145 | -434.944 | 0.653 | 0.051 | 0 | 0.678 |
| EM | Logical memory 1 | -377.806 | 0.03 | -205.082 | 0.007 | -308.57 | -474.475 | -142.666 | **0.000***** | 0.316 | 0.227 |
|  | Logical memory 2 | -236.665 | 0.022 | -193.572 | 0.012 | -221.538 | -318.807 | -124.269 | **0.000***** | 0 | 0.679 |
|  | Faces 1 | -85.698 | 0.012 | -134.584 | 0.024 | -94.815 | -153.364 | -36.267 | **0.002**** | 0 | 0.524 |
|  | Faces 2 | -74.545 | 0.009 | -60.495 | 0.005 | -71.837 | -129.448 | -14.226 | 0.015* | 0 | 0.85 |
| WM | SWM (errors) | 259.21 | 0.005 | 49.944 | 0 | 200.546 | -45.339 | 446.43 | 0.11 | 0 | 0.454 |
|  | SWM (strategy) | 74.282 | 0.004 | -51.013 | 0.002 | 39.889 | -45.908 | -45.908 | 0.362 | 0.387 | 0.201 |
|  | Letter-number sequencing | -40.847 | 0.006 | -29.095 | 0.002 | -36.605 | -69.831 | -3.378 | 0.031* | 0 | 0.739 |
| Attention | SART reaction time | 1451.099 | 0.007 | 4360.635 | 0.047 | 2048.904 | -255.171 | 4352.979 | 0.081 | 0.289 | 0.236 |
|  | CPT d’Prime 2 digit | -10.559 | 0.003 | -8.797 | 0.003 | -9.986 | -26.034 | 6.061 | 0.223 | 0 | 0.92 |
|  | CPT d’Prime 3 digit | -2.927 | 0 | 3.221 | 0 | -1.246 | -16.494 | 14.002 | 0.873 | 0 | 0.725 |
|  | CPT d’Prime 4 digit | 6.205 | 0.002 | 1.005 | 0 | 5.034 | -7.721 | 17.79 | 0.439 | 0 | 0.739 |
| Social Cognition | Eyes | 33.373 | 0.002 | 20.241 | 0 | 27.701 | -39.15 | 94.553 | 0.417 | 0 | 0.849 |
|  | Hint | -43.163 | 0.009 | 18.881 | 0.002 | -23.224 | -80.013 | 33.566 | 0.423 | 0.377 | 0.205 |
|  | Externalising Bias | 20.474 | 0.001 | 8.051 | 0 | 18.604 | -30.143 | 67.352 | 0.454 | 0 | 0.858 |
|  | Personalising Bias | 2.324 | 0.003 | 2.046 | 0.013 | -0.546 | -7.551 | 6.458 | 0.878 | 0.628 | 0.101 |

**p*<0.05; ***p*<0.01; ****p*<0.0001. Results in bold indicate significance after correction for multiple testing across 5 cognitive domains.

*MIR137*, microRNA 137; IQ, intelligence quotient; WTAR, Wechsler Test of Adult Reading; DM, declarative memory; WM, working memory; SART, Sustained Attention to Response Task; CPT, Continuous Performance Task. MiR-137 polygene scores were thresholded at *p*=10^-5^, *p*=0.05 and *p*=0.5.

**Supplementary Table 1.2:** Inverse variance meta-analysis results at *p*=0.05 *MIR137* polygene risk score threshold for whole sample

|  |  | Sample A |  | Sample B |  | Combined Sample | 95% CI | Combined Sample |  | Heterogeneity | |
| --- | --- | --- | --- | --- | --- | --- | --- | --- | --- | --- | --- |
|  | Neuropsychological Variable | (n=597) | r^2^ | (n=403) | r^2^ | B | Lower | Upper | *p* | I^2^ | *p* |
|  |  | B |  | B |  |  |  |  |  |  |  |
| IQ | Pre-morbid IQ (WTAR) | -871.584 | 0.003 | -862.148 | 0.003 | -503.302 | -1165.205 | 158.601 | 0.136 | 0.433 | 0.184 |
|  | Verbal IQ | -3101.487 | 0.008 | -6565.796 | 0.032 | -4618.646 | -7987.354 | -1249.938 | 0.035* | 0.573 | 0.126 |
|  | Performance IQ | -3153.954 | 0.009 | -4808.165 | 0.016 | -3515.312 | -5765.215 | -1265.41 | **0.002**** | 0 | 0.552 |
|  | Full-scale IQ | -3604.812 | 0.012 | -4983.026 | 0.02 | -3959.537 | -6193.356 | -1725.718 | **0.001**** | 0 | 0.597 |
| DM | Logical memory 1 | -3588.765 | 0.026 | -3748.627 | 0.021 | -3641.81 | -5037.08 | -2246.541 | **0.000***** | 0 | 0.916 |
|  | Logical memory 2 | -2491.725 | 0.024 | -3278.128 | 0.03 | -2747.838 | -3757.958 | -1737.718 | **0.000***** | 0 | 0.475 |
|  | Faces 1 | -989.82 | 0.015 | -1145.821 | 0.013 | -1013.981 | -1626.683 | -401.278 | **0.002**** | 0 | 0.857 |
|  | Faces 2 | -900.844 | 0.012 | -227.323 | 0.001 | -791.795 | -1394.458 | -189.133 | 0.015* | 0 | 0.42 |
| WM | SWM (errors) | 4719.332 | 0.015 | 783.902 | 0 | 3260.415 | -465.077 | 6985.908 | 0.086 | 0.447 | 0.179 |
|  | SWM (strategy) | 910.596 | 0.005 | 161.021 | 0 | 710.783 | -186.172 | 1607.738 | 0.12 | 0 | 0.469 |
|  | Letter-number sequencing | -613.169 | 0.012 | -637.372 | 0.01 | -621.413 | -967.522 | -275.304 | 0.031* | 0 | 0.948 |
| Attention | SART reaction time | 19607.843 | 0.012 | 24740.421 | 0.011 | 19926.956 | 6182.706 | 33671.206 | **0.004**** | 0 | 0.86 |
|  | CPT d’Prime 2 digit | -21.517 | 0 | 19.276 | 0 | -9.181 | -181.359 | 162.997 | 0.917 | 0 | 0.831 |
|  | CPT d’Prime 3 digit | 20.517 | 0 | 16.751 | 0 | 19.569 | -142.902 | 182.039 | 0.813 | 0 | 0.984 |
|  | CPT d’Prime 4 digit | 53.913 | 0.002 | 15.335 | 0 | 46.054 | -89.501 | 181.609 | 0.505 | 0 | 0.822 |
| Social Cognition | Eyes | 11.977 | 0 | -173.013 | 0 | -61.41 | -748.152 | 625.332 | 0.861 | 0 | 0.796 |
|  | Hint | -735.969 | 0.023 | -13.119 | 0 | -503.302 | -1165.205 | 158.601 | 0.136 | 0.433 | 0.184 |
|  | Externalising Bias | 105.658 | 0 | 712.51 | 0.008 | 194.019 | -327.109 | 715.147 | 0.466 | 0 | 0.421 |
|  | Personalising Bias | 6.564 | 0 | -71.31 | 0.022 | -23.491 | -97.795 | 50.812 | 0.535 | 0.615 | 0.107 |

**p*<0.05; ***p*<0.01; ****p*<0.0001. Results in bold indicate significance after correction for multiple testing across 5 cognitive domains.

*MIR137*, microRNA 137; IQ, intelligence quotient; WTAR, Wechsler Test of Adult Reading; DM, declarative memory; WM, working memory; SART, Sustained Attention to Response Task; CPT, Continuous Performance Task. MiR-137 polygene scores were thresholded at *p*=10^-5^, *p*=0.05 and *p*=0.5.

**Supplementary Table 1.3:** Inverse variance meta-analysis results at *p*=0.5 *MIR137* polygene risk score threshold for whole sample

|  |  | Sample A |  | Sample B |  | Combined Sample | 95% CI | Combined Sample |  | Heterogeneity | |
| --- | --- | --- | --- | --- | --- | --- | --- | --- | --- | --- | --- |
|  | Neuropsychological Variable | (n=597) | r^2^ | (n=403) | r^2^ | B | Lower | Upper | *p* | I^2^ | *p* |
|  |  | B |  | B |  |  |  |  |  |  |  |
| IQ | Pre-morbid IQ (WTAR) | -999.72 | 0 | -2912.471 | 0.004 | -1671.986 | -5226.713 | 1882.74 | 0.357 | 0 | 0.869 |
|  | Verbal IQ | -7450.949 | 0.004 | -23532.628 | 0.039 | -15034.193 | -30768.408 | 700.022 | 0.035* | 0.791 | 0.029 |
|  | Performance IQ | -8936.811 | 0.007 | -12369.362 | 0.01 | -9699.454 | -17083.868 | -2315.041 | **0.010*** | 0 | 0.705 |
|  | Full-scale IQ | -9145.101 | 0.007 | -13691.762 | 0.021 | -11042.282 | -18376.914 | -3707.651 | **0.003**** | 0 | 0.395 |
| DM | Logical memory 1 | -9733.429 | 0.018 | -12634.313 | 0.023 | -10669.811 | -11909.44 | -6125.766 | **0.000***** | 0 | 0.579 |
|  | Logical memory 2 | -6958.005 | 0.017 | -10760.329 | 0.031 | -8330.306 | -11909.44 | -4751.172 | **0.000***** | 0.131 | 0.283 |
|  | Faces 1 | -2419.292 | 0.008 | -2881.015 | 0.008 | -2492.285 | -4493.001 | -491.569 | **0.002**** | 0 | 0.869 |
|  | Faces 2 | -1758.938 | 0.004 | -407.728 | 0 | -1534.968 | -3504.421 | 434.485 | 0.015* | 0 | 0.869 |
| WM | SWM (errors) | 12307.355 | 0.01 | 2094.429 | 0 | 9254.991 | 91.947 | 18418.036 | 0.048* | 0.132 | 0.283 |
|  | SWM (strategy) | 1323.068 | 0.001 | 98.719 | 0 | 994.328 | -1929.699 | 3918.354 | 0.505 | 0 | 0.716 |
|  | Letter-number sequencing | -1505.131 | 0.007 | -2151.773 | 0.011 | -1732.88 | -2867.928 | -597.833 | 0.031* | 0 | 0.594 |
| Attention | SART reaction time | 49412.521 | 0.007 | -6591.584 | 0 | 45561.862 | -1929.699 | 3918.354 | 0.505 | 0 | 0.716 |
|  | CPT d’Prime 2 digit | 192.79 | 0.001 | -24.815 | 0 | 128.901 | -430.676 | 688.477 | 0.652 | 0 | 0.729 |
|  | CPT d’Prime 3 digit | 285.963 | 0.002 | -174.303 | 0.001 | 243.74 | -204.455 | 691.934 | 0.286 | 0 | 0.839 |
|  | CPT d’Prime 4 digit | 213.494 | 0.002 | 294.486 | 0.003 | 229.518 | -213.062 | 672.099 | 0.309 | 0 | 0.886 |
| Social Cognition | Eyes | 642.104 | 0.001 | -11010.951 | 0.001 | -96.271 | -2371.226 | 2178.684 | 0.934 | 0 | 0.458 |
|  | Hint | -2249.994 | 0.021 | -77.085 | 0 | -1553.49 | -3541.015 | 434.036 | 0.126 | 0.398 | 0.198 |
|  | Externalising Bias | -131.267 | 0 | 2188.519 | 0.008 | 211.583 | -1473.479 | 1896.644 | 0.806 | 0 | 0.338 |
|  | Personalising Bias | 16.71 | 0 | -201.361 | 0.017 | -59.643 | -263.523 | 144.236 | 0.566 | 0.491 | 0.161 |

**p*<0.05; ***p*<0.01; ****p*<0.0001. Results in bold indicate significance after correction for multiple testing across 5 cognitive domains.

*MIR137*, microRNA 137; IQ, intelligence quotient; WTAR, Wechsler Test of Adult Reading; DM, declarative memory; WM, working memory; SART, Sustained Attention to Response Task; CPT, Continuous Performance Task. MiR-137 polygene scores were thresholded at *p*=10^-5^, *p*=0.05 and *p*=0.5.

**Supplementary Table 2.1:** Inverse variance meta-analysis results at *p*=10^-5^ *MIR137* polygene risk score threshold in the broad psychosis group

|  |  | Sample A |  | Sample B |  | Combined Sample | 95% CI | Combined Sample |  | Heterogeneity | |
| --- | --- | --- | --- | --- | --- | --- | --- | --- | --- | --- | --- |
|  | Neuropsychological Variable | (n=505) | r^2^ | (n=303) | r^2^ | B | Lower | Upper | p | I^2^ | *p* |
|  |  | B |  | B |  |  |  |  |  |  |  |
| IQ | Pre-morbid IQ (WTAR) | 45.92 | 0.001 | 66.223 | 0.002 | 51.137 | -78.596 | 180.871 | 0.44 | 0 | 0.893 |
|  | Verbal IQ | 57.78 | 0 | -397.252 | 0.016 | -149.585 | -593.756 | 294.586 | 0.509 | 0.739 | 0.05 |
|  | Performance IQ | 133.155 | 0.002 | -54.546 | 0 | 96.53 | -133.846 | 326.907 | 0.412 | 0 | 0.527 |
|  | Full-scale IQ | 86.943 | 0.001 | -257.1 | 0.007 | -5.273 | -303.95 | 293.404 | 0.972 | 0.249 | 0.249 |
| DM | Logical memory 1 | -188.558 | 0.01 | -50.064 | 0.001 | -141.378 | -273.958 | -8.798 | 0.037* | 0 | 0.332 |
|  | Logical memory 2 | -106.25 | 0.006 | -70.691 | 0.002 | -94.412 | -187.955 | -0.869 | 0.048* | 0 | 0.726 |
|  | Faces 1 | -24.885 | 0.001 | -37.345 | 0.002 | -26.605 | -93.315 | 40.105 | 0.434 | 0 | 0.9 |
|  | Faces 2 | -52.647 | 0.004 | -1.015 | 0 | -47.144 | -116.361 | 22.074 | 0.182 | 0 | 0.652 |
| WM | SWM (errors) | 103.522 | 0.001 | -218.239 | 0.004 | 24.264 | -262.65 | 311.178 | 0.868 | 0 | 0.344 |
|  | SWM (strategy) | 62.541 | 0.002 | -106.909 | 0.011 | -8.7 | -172.642 | 155.241 | 0.917 | 0.579 | 0.123 |
|  | Letter-number sequencing | -3.455 | 0 | 4.412 | 0 | -0.709 | -35.114 | 33.697 | 0.968 | 0 | 0.831 |
| Attention | SART reaction time | 522.913 | 0.001 | 1732.656 | 0.01 | 639.848 | -836.394 | 2116.091 | 0.396 | 0 | 0.635 |
|  | CPT d’Prime 2 digit | -10.559 | 0.003 | 2.332 | 0 | -7.147 | -23.895 | 9.601 | 0.403 | 0 | 0.506 |
|  | CPT d’Prime 3 digit | -2.927 | 0 | 13.389 | 0.007 | 1.017 | -14.56 | 16.594 | 0.898 | 0 | 0.379 |
|  | CPT d’Prime 4 digit | 6.205 | 0.002 | 7.963 | 0.004 | 6.62 | -6.045 | 19.285 | 0.306 | 0 | 0.908 |
| Social Cognition | Eyes | 113.044 | 0.018 | 43.059 | 0.002 | 72.259 | -14.967 | 159.486 | 0.104 | 0 | 0.438 |
|  | Hint | -34.294 | 0.004 | 75.982 | 0.019 | 3.519 | -99.076 | 106.114 | 0.946 | 0.546 | 0.138 |
|  | Externalising Bias | -3.003 | 0 | -47.169 | 0.004 | -8.057 | -67.309 | 51.195 | 0.79 | 0 | 0.642 |
|  | Personalising Bias | 4.397 | 0.01 | -2.198 | 0.002 | 3.126 | -1.974 | 8.225 | 0.23 | 0.084 | 0.296 |

**p*<0.05; ***p*<0.01; ****p*<0.0001. Results in bold indicate significance after correction for multiple testing across 5 cognitive domains.

*MIR137*, microRNA 137; IQ, intelligence quotient; WTAR, Wechsler Test of Adult Reading; DM, declarative memory; WM, working memory; SART, Sustained Attention to Response Task; CPT, Continuous Performance Task. MiR-137 polygene scores were thresholded at *p*=10^-5^, *p*=0.05 and *p*=0.5.

**Supplementary Table 2.2:** Inverse variance meta-analysis results at *p*=0.05 *MIR137* polygene risk score threshold in the broad psychosis group

|  |  | Sample A |  | Sample B |  | Combined Sample | 95% CI | Combined Sample |  | Heterogeneity | |
| --- | --- | --- | --- | --- | --- | --- | --- | --- | --- | --- | --- |
|  | Neuropsychological Variable | (n=505) | r^2^ | (n=303) | r^2^ | B | Lower | Upper | *p* | I^2^ | *p* |
|  |  | B |  | B |  |  |  |  |  |  |  |
| IQ | Pre-morbid IQ (WTAR) | 298.166 | 0 | 143.503 | 0 | 262.734 | -1067.898 | 1593.366 | 0.699 | 0 | 0.924 |
|  | Verbal IQ | -532.936 | 0 | -5085.518 | 0.021 | -2525.022 | -6951.56 | 1901.516 | 0.264 | 0.699 | 0.068 |
|  | Performance IQ | -516.508 | 0 | -1015.211 | 0.001 | -601.069 | -2978.267 | 1776.129 | 0.62 | 0 | 0.877 |
|  | Full-scale IQ | -809.894 | 0.001 | -2231.912 | 0.004 | -1044.144 | -3410.598 | 1322.31 | 0.387 | 0 | 0.662 |
| DM | Logical memory 1 | -967.353 | 0.003 | -1252.287 | 0.003 | -1056.308 | -2444.234 | 331.618 | 0.136 | 0 | 0.852 |
|  | Logical memory 2 | -650.908 | 0.002 | -1399.539 | 0.008 | -877.281 | -1854.245 | 99.683 | 0.078 | 0 | 0.49 |
|  | Faces 1 | -271.363 | 0.001 | 762.413 | 0.006 | -158.523 | -857.744 | 540.699 | 0.657 | 0 | 0.366 |
|  | Faces 2 | -655.896 | 0.006 | 3.595 | 0 | -599.771 | -1321.289 | 121.747 | 0.103 | 0 | 0.617 |
| WM | SWM (errors) | 3085.254 | 0.006 | -2411.46 | 0.004 | 978.073 | -4259.968 | 6216.115 | 0.714 | 0.568 | 0.128 |
|  | SWM (strategy) | 596.45 | 0.002 | -532.261 | 0.002 | 264.531 | -779.147 | 1308.209 | 0.619 | 0 | 0.334 |
|  | Letter-number sequencing | -258.632 | 0.002 | -86.468 | 0 | -203.283 | -565.977 | 159.411 | 0.272 | 0 | 0.664 |
| Attention | SART reaction time | 7928.916 | 0.002 | -12030.619 | 0.003 | 6609.674 | -8448.006 | 21667.354 | 0.39 | 0 | 0.519 |
|  | CPT d’Prime 2 digit | -21.517 | 0 | 127.131 | 0.004 | 12.585 | -168.378 | 193.547 | 0.892 | 0 | 0.498 |
|  | CPT d’Prime 3 digit | 20.517 | 0 | 75.466 | 0.002 | 31.8 | -135.636 | 199.236 | 0.71 | 0 | 0.795 |
|  | CPT d’Prime 4 digit | 53.913 | 0.002 | 122.662 | 0.006 | 67.663 | -68.209 | 203.534 | 0.329 | 0 | 0.692 |
| Social Cognition | Eyes | 596.555 | 0.005 | -29.851 | 0 | 265.737 | -645.323 | 1176.796 | 0.568 | 0 | 0.501 |
|  | Hint | -707.137 | 0.016 | 305.168 | 0.002 | -460.454 | -1312.229 | 391.322 | 0.289 | 0.306 | 0.23 |
|  | Externalising Bias | -164.459 | 0.001 | 612.601 | 0.005 | -86.06 | -730.9 | 558.78 | 0.794 | 0 | 0.477 |
|  | Personalising Bias | 27.631 | 0.003 | -96.679 | 0.033 | -20.153 | -138.674 | 98.367 | 0.739 | 0.669 | 0.082 |

**p*<0.05; ***p*<0.01; ****p*<0.0001. Results in bold indicate significance after correction for multiple testing across 5 cognitive domains.

*MIR137*, microRNA 137; IQ, intelligence quotient; WTAR, Wechsler Test of Adult Reading; DM, declarative memory; WM, working memory; SART, Sustained Attention to Response Task; CPT, Continuous Performance Task. MiR-137 polygene scores were thresholded at *p*=10^-5^, *p*=0.05 and *p*=0.5.

**Supplementary Table 2.3:** Inverse variance meta-analysis results at *p*=0.5 *MIR137* polygene risk score threshold in the broad psychosis group

|  |  | Sample A |  | Sample B |  | Combined Sample | 95% CI | Combined Sample |  | Heterogeneity | |
| --- | --- | --- | --- | --- | --- | --- | --- | --- | --- | --- | --- |
|  | Neuropsychological Variable | (n=505) | r^2^ | (n=303) | r^2^ | B | Lower | Upper | *p* | I^2^ | *p* |
|  |  | B |  | B |  |  |  |  |  |  |  |
| IQ | Pre-morbid IQ (WTAR) | 3005.744 | 0.003 | 621.128 | 0 | 2422.852 | -1883.183 | 6728.887 | 0.27 | 0 | 0.641 |
|  | Verbal IQ | 1439.713 | 0 | -15872.619 | 0.02 | -6466.8 | -23368.831 | 10435.231 | 0.453 | 0.784 | 0.032 |
|  | Performance IQ | -895.487 | 0 | -1763.167 | 0 | -1052.248 | -8769.259 | 6664.762 | 0.789 | 0 | 0.932 |
|  | Full-scale IQ | 73.163 | 0 | -5734.062 | 0.003 | -939.531 | -8622.939 | 6743.877 | 0.811 | 0 | 0.574 |
| DM | Logical memory 1 | -1359.511 | 0 | -3219.885 | 0.002 | -1961.2 | -6460.213 | 2537.813 | 0.393 | 0 | 0.705 |
|  | Logical memory 2 | -1079.227 | 0.001 | -4127.135 | 0.007 | -2034.307 | -5203.535 | 1134.922 | 0.208 | 0 | 0.382 |
|  | Faces 1 | -222.295 | 0 | 3037.131 | 0.011 | 160.314 | -2088.187 | 2408.815 | 0.889 | 0 | 0.36 |
|  | Faces 2 | -786.147 | 0.001 | -76.167 | 0 | -722.168 | -3053.127 | 1608.791 | 0.544 | 0 | 0.864 |
| WM | SWM (errors) | 6637.018 | 0.003 | -9554.2 | 0.006 | 691.051 | -14606.455 | 15988.557 | 0.929 | 0.496 | 0.159 |
|  | SWM (strategy) | -392.859 | 0 | -3265.413 | 0.009 | -1260.725 | -4613.016 | 2091.567 | 0.461 | 0 | 0.441 |
|  | Letter-number sequencing | -456.447 | 0.001 | -500.548 | 0.001 | -471.121 | -1652.905 | 710.662 | 0.435 | 0 | 0.973 |
| Attention | SART reaction time | 4075.067 | 0 | -107201.6 | 0.026 | -16981.979 | -102409.4 | 68445.447 | 0.697 | 0.271 | 0.241 |
|  | CPT d’Prime 2 digit | 192.79 | 0.001 | 140.07 | 0.001 | 180.9 | -405.019 | 766.82 | 0.545 | 0 | 0.941 |
|  | CPT d’Prime 3 digit | 285.963 | 0.002 | -157.533 | 0.001 | 196.793 | -344.955 | 738.542 | 0.476 | 0 | 0.52 |
|  | CPT d’Prime 4 digit | 213.494 | 0.002 | 445.699 | 0.008 | 259.807 | -182.332 | 701.946 | 0.249 | 0 | 0.681 |
| Social Cognition | Eyes | 2434.672 | 0.007 | -860.56 | 0.001 | 603.999 | -2605.276 | 3813.274 | 0.712 | 0.122 | 0.286 |
|  | Hint | -2188.762 | 0.016 | 852.824 | 0.002 | -1521.454 | -3988.499 | 945.591 | 0.227 | 0.235 | 0.253 |
|  | Externalising Bias | -857.528 | 0.002 | 3115.134 | 0.014 | -57.443 | -3180.086 | 3065.2 | 0.971 | 0.248 | 0.249 |
|  | Personalising Bias | 83.244 | 0.003 | -349.475 | 0.042 | -92.456 | -508.958 | 324.046 | 0.664 | 0.728 | 0.055 |

**p*<0.05; ***p*<0.01; ****p*<0.0001. Results in bold indicate significance after correction for multiple testing across 5 cognitive domains.

*MIR137*, microRNA 137; IQ, intelligence quotient; WTAR, Wechsler Test of Adult Reading; DM, declarative memory; WM, working memory; SART, Sustained Attention to Response Task; CPT, Continuous Performance Task. MiR-137 polygene scores were thresholded at *p*=10^-5^, *p*=0.05 and *p*=0.5.

**Supplementary Table 3.1:** Inverse variance meta-analysis results at *p*=10^-5^ *MIR137* polygene risk score threshold in narrow psychosis (SZ/SZA) sample

|  |  | Sample A |  | Sample B |  | Combined Sample | 95% CI | Combined Sample |  | Heterogeneity | |
| --- | --- | --- | --- | --- | --- | --- | --- | --- | --- | --- | --- |
|  | Neuropsychological Variable | (n=396) | r^2^ | (n=189) | r^2^ | B | Lower | Upper | *p* | I^2^ | *p* |
|  |  | B |  | B |  |  |  |  |  |  |  |
| IQ | Pre-morbid IQ (WTAR) | 16.846 | 0 | 124.98 | 0.005 | 35.526 | -115.109 | 186.16 | 0.644 | 0 | 0.595 |
|  | Verbal IQ | -16.182 | 0 | -238.345 | 0.006 | -81.249 | -324.31 | 161.813 | 0.512 | 0 | 0.415 |
|  | Performance IQ | 53.439 | 0 | -71.388 | 0.001 | 35.821 | -216.447 | 288.09 | 0.781 | 0 | 0.736 |
|  | Full-scale IQ | -2.551 | 0 | -319.491 | 0.012 | -55.424 | -308.228 | 197.379 | 0.667 | 0 | 0.36 |
| DM | Logical memory 1 | -192.663 | 0.011 | -53.064 | 0.001 | -154.196 | -310.516 | 2.124 | 0.053 | 0 | 0.434 |
|  | Logical memory 2 | -96.835 | 0.006 | -42.153 | 0.001 | -81.441 | -189.875 | 26.992 | 0.141 | 0 | 0.657 |
|  | Faces 1 | -41.327 | 0.003 | 3.609 | 0 | -36.098 | -112.019 | 39.824 | 0.351 | 0 | 0.71 |
|  | Faces 2 | -61.083 | 0.006 | 22.255 | 0.001 | -53.012 | -129.36 | 23.336 | 0.174 | 0 | 0.527 |
| WM | SWM (errors) | 223.794 | 0.004 | 51.037 | 0 | 199.006 | -141.962 | 539.973 | 0.253 | 0 | 0.728 |
|  | SWM (strategy) | 107.023 | 0.007 | 29.868 | 0.001 | 93.184 | -26.363 | 212.732 | 0.127 | 0 | 0.627 |
|  | Letter-number sequencing | -16.282 | 0.001 | -25.401 | 0.002 | -18.718 | -59.273 | 21.837 | 0.366 | 0 | 0.845 |
| Attention | SART reaction time | 459.894 | 0.001 | 3495.623 | 0.034 | 1143.461 | -1341.802 | 3628.724 | 0.367 | 0.279 | 0.239 |
|  | CPT d’Prime 2 digit | -10.037 | 0.003 | 11.775 | 0.004 | -6.779 | -26.58 | 13.022 | 0.502 | 0 | 0.442 |
|  | CPT d’Prime 3 digit | -0.359 | 0 | 17.605 | 0.009 | 2.093 | -15.427 | 19.612 | 0.815 | 0 | 0.49 |
|  | CPT d’Prime 4 digit | 10.087 | 0.007 | -2.784 | 0 | 8.311 | -5.368 | 21.991 | 0.234 | 0 | 0.525 |
| Social Cognition | Eyes | 93.619 | 0.011 | -50.706 | 0.002 | 23.254 | -118.138 | 164.646 | 0.747 | 0.416 | 0.191 |
|  | Hint | -40.655 | 0.006 | 126.482 | 0.043 | 26.496 | -134.103 | 187.095 | 0.746 | 0.719 | 0.059 |
|  | Externalising Bias | 7.955 | 0 | 19.219 | 0.001 | 9.459 | -57.884 | 76.802 | 0.783 | 0 | 0.911 |
|  | Personalising Bias | 4.267 | 0.01 | -2.022 | 0.002 | 3.284 | -1.806 | 8.373 | 0.206 | 0 | 0.379 |

**p*<0.05; ***p*<0.01; ****p*<0.0001. Results in bold indicate significance after correction for multiple testing across 5 cognitive domains.

*MIR137*, microRNA 137; IQ, intelligence quotient; WTAR, Wechsler Test of Adult Reading; DM, declarative memory; WM, working memory; SART, Sustained Attention to Response Task; CPT, Continuous Performance Task. MiR-137 polygene scores were thresholded at *p*=10^-5^, *p*=0.05 and *p*=0.5.

**Supplementary Table 3.2:** Inverse variance meta-analysis results at *p*=0.05 *MIR137* polygene risk score threshold in narrow psychosis (SZ/SZA) sample

|  |  | Sample A |  | Sample B |  | Combined Sample | 95% CI | Combined Sample |  | Heterogeneity | |
| --- | --- | --- | --- | --- | --- | --- | --- | --- | --- | --- | --- |
|  | Neuropsychological Variable | (n=396) | r^2^ | (n=189) | r^2^ | B | Lower | Upper | *p* | I^2^ | *p* |
|  |  | B |  | B |  |  |  |  |  |  |  |
| IQ | Pre-morbid IQ (WTAR) | -232.426 | 0 | 700.798 | 0.002 | -78.746 | -1634.617 | 1477.126 | 0.921 | 0 | 0.663 |
|  | Verbal IQ | -1695.428 | 0.003 | -3474.254 | 0.011 | -2194.956 | -4716.116 | 326.205 | 0.088 | 0 | 0.534 |
|  | Performance IQ | -1624.525 | 0.003 | 649.413 | 0 | -1334.701 | -3962.492 | 1293.09 | 0.319 | 0 | 0.572 |
|  | Full-scale IQ | -2119.808 | 0.005 | -1510.596 | 0.002 | -2029.441 | -4661.461 | 602.579 | 0.131 | 0 | 0.872 |
| DM | Logical memory 1 | -1709.965 | 0.008 | -627.504 | 0.001 | -1427.757 | -3076.61 | 221.096 | 0.09 | 0 | 0.572 |
|  | Logical memory 2 | -967.827 | 0.005 | -1012.934 | 0.004 | -979.725 | -2123.357 | 163.907 | 0.093 | 0 | 0.973 |
|  | Faces 1 | -434.86 | 0.003 | 907.899 | 0.008 | -282.244 | -1117.555 | 553.067 | 0.508 | 0.024 | 0.312 |
|  | Faces 2 | -924.522 | 0.012 | 79.607 | 0 | -837.112 | -1632.802 | -41.422 | 0.039* | 0 | 0.486 |
| WM | SWM (errors) | 4771.004 | 0.015 | -1877.751 | 0.002 | 2868.582 | -3020.948 | 8758.112 | 0.34 | 0.4 | 0.197 |
|  | SWM (strategy) | 1217.76 | 0.008 | -115.169 | 0 | 976.231 | -272.539 | 2225.002 | 0.125 | 0 | 0.42 |
|  | Letter-number sequencing | -572.332 | 0.012 | -298.209 | 0.002 | -469.625 | -896.628 | -42.622 | 0.031* | 0 | 0.648 |
| Attention | SART reaction time | 6296.351 | 0.001 | -21407.655 | 0.009 | 3919.355 | -12999.778 | 20838.489 | 0.65 | 0 | 0.369 |
|  | CPT d’Prime 2 digit | -5.904 | 0 | 409.414 | 0.036 | 119.454 | -254.22 | 493.129 | 0.531 | 0.446 | 0.179 |
|  | CPT d’Prime 3 digit | 25.255 | 0 | 252.34 | 0.015 | 53.996 | -133.014 | 241.005 | 0.571 | 0 | 0.429 |
|  | CPT d’Prime 4 digit | 81.19 | 0.004 | 226.716 | 0.021 | 99.509 | -45.017 | 244.035 | 0.177 | 0 | 0.513 |
| Social Cognition | Eyes | 288.392 | 0.001 | -201.895 | 0 | 80.789 | -1033.817 | 1195.395 | 0.887 | 0 | 0.662 |
|  | Hint | -949.97 | 0.027 | 636.5 | 0.01 | -364.021 | -1864.711 | 1136.67 | 0.634 | 0.636 | 0.097 |
|  | Externalising Bias | -111.55 | 0 | 359.521 | 0.002 | -53.085 | -779.275 | 673.105 | 0.886 | 0 | 0.675 |
|  | Personalising Bias | 33.702 | 0.006 | -120.791 | 0.049 | -29.861 | -178.867 | 119.145 | 0.694 | 0.746 | 0.047 |

**p*<0.05; ***p*<0.01; ****p*<0.0001. Results in bold indicate significance after correction for multiple testing across 5 cognitive domains.

*MIR137*, microRNA 137; IQ, intelligence quotient; WTAR, Wechsler Test of Adult Reading; DM, declarative memory; WM, working memory; SART, Sustained Attention to Response Task; CPT, Continuous Performance Task. MiR-137 polygene scores were thresholded at *p*=10^-5^, *p*=0.05 and *p*=0.5.

**Supplementary Table 3.3:** Inverse variance meta-analysis results at p=0.5 *MIR137* polygene risk score threshold in the narrow psychosis (SZ/SZA) sample

|  |  | Sample A |  | Sample B |  | Combined Sample | 95% CI | Combined Sample |  | Heterogeneity | |
| --- | --- | --- | --- | --- | --- | --- | --- | --- | --- | --- | --- |
|  | Neuropsychological Variable | (n=396) | r^2^ | (n=189) | r^2^ | B | Lower | Upper | *p* | I^2^ | *p* |
|  |  | B |  | B |  |  |  |  |  |  |  |
| IQ | Pre-morbid IQ (WTAR) | 1175.502 | 0 | 239.364 | 0 | 1012.221 | -4055.79 | 6080.232 | 0.695 | 0 | 0.891 |
|  | Verbal IQ | -2368.42 | 0.001 | -12238.481 | 0.013 | -5225.175 | -13998.132 | 3547.782 | 0.243 | 0.071 | 0.3 |
|  | Performance IQ | -4055.363 | 0.002 | -512.755 | 0 | -3588.936 | -12192.138 | 5014.265 | 0.414 | 0 | 0.785 |
|  | Full-scale IQ | -3627.092 | 0.001 | -7184.125 | 0.005 | -4172.832 | -12784.997 | 4439.333 | 0.342 | 0 | 0.77 |
| DM | Logical memory 1 | -3682.667 | 0.003 | -2299.523 | 0.001 | -3310.806 | -8695.893 | 2074.281 | 0.228 | 0 | 0.823 |
|  | Logical memory 2 | -2078.687 | 0.002 | -3188.532 | 0.004 | -2380.265 | -6116.649 | 1356.119 | 0.212 | 0 | 0.796 |
|  | Faces 1 | -1240.027 | 0.002 | 4023.376 | 0.015 | -23.485 | -4372.298 | 4325.328 | 0.992 | 0.322 | 0.225 |
|  | Faces 2 | 578.598 | 0.005 | -1936.041 | 0 | -1721.237 | -4322.981 | 880.508 | 0.195 | 0 | 0.596 |
| WM | SWM (errors) | 11422.479 | 0.008 | -9312.672 | 0.005 | 6013.09 | -11832.595 | 23858.776 | 0.509 | 0.332 | 0.221 |
|  | SWM (strategy) | 1176.598 | 0.001 | -4379.361 | 0.012 | 86.097 | -4238.982 | 4411.177 | 0.969 | 0.048 | 0.305 |
|  | Letter-number sequencing | -1338.38 | 0.006 | -1260.718 | 0.004 | -1318.061 | -2723.267 | 87.145 | 0.066 | 0 | 0.962 |
| Attention | SART reaction time | -1342.044 | 0 | -142293.78 | 0.036 | -42396.873 | -167914.94 | 83121.19 | 0.508 | 0.496 | 0.159 |
|  | CPT d’Prime 2 digit | 103.996 | 0 | 735.339 | 0.01 | 187.155 | -510.68 | 884.991 | 0.599 | 0 | 0.549 |
|  | CPT d’Prime 3 digit | 154.725 | 0.001 | 444.357 | 0.004 | 188.847 | -425.574 | 803.269 | 0.547 | 0 | 0.766 |
|  | CPT d’Prime 4 digit | 223.372 | 0.003 | 930.969 | 0.032 | 308.444 | -162.11 | 778.997 | 0.199 | 0 | 0.338 |
| Social Cognition | Eyes | 1561.393 | 0.003 | -1688.613 | 0.002 | 48.651 | -3608.855 | 3706.157 | 0.979 | 0 | 0.385 |
|  | Hint | -3335.855 | 0.032 | 2840.818 | 0.017 | -837.962 | -6779.336 | 5103.412 | 0.782 | 0.739 | 0.05 |
|  | Externalising Bias | -1015.558 | 0.003 | 965.132 | 0.001 | -783.923 | -3139.039 | 1571.193 | 0.514 | 0 | 0.596 |
|  | Personalising Bias | 116.958 | 0.006 | -544.335 | 0.089 | -179.385 | -823.943 | 465.174 | 0.585 | 0.852 | 0.009 |

**p*<0.05; ***p*<0.01; ****p*<0.0001. Results in bold indicate significance after correction for multiple testing across 5 cognitive domains.

*MIR137*, microRNA 137; IQ, intelligence quotient; WTAR, Wechsler Test of Adult Reading; DM, declarative memory; WM, working memory; SART, Sustained Attention to Response Task; CPT, Continuous Performance Task. MiR-137 polygene scores were thresholded at *p*=10^-5^, *p*=0.05 and *p*=0.5.

**Supplementary Table 4:** *MIR137* polygenic score regression analysis for each neuropsychological variable in all cases and controls (n=988) (regardless of genotyping platform).

|  | Neuropsychological | p=10^-5^ |  | p=0.05 |  | p=0.5 |  |
| --- | --- | --- | --- | --- | --- | --- | --- |
|  | Variable | Standardised β | r^2^ (*p*) | Standardised β | r^2^ (*p*) | Standardised β | r^2^ (*p*) |
| IQ | Pre-morbid IQ (WTAR)  Verbal IQ  Performance IQ  Full-scale IQ | -0.033  **-0.085**  -0.051  -0.073 | 0.001 (0.349)  **0.007 (0.007)****  0.003 (0.133)  0.005 (0.031)* | -0.055  **-0.123**  **-0.109**  **-0.122** | 0.003 (0.114)  **0.015 (0.000)*****  **0.012 (0.001)****  **0.015 (0.000)***** | -0.030  **-0.114**  **-0.093**  **-0.105** | 0.001 (0.383)  **0.013 (0.000)*****  **0.009 (0.006)****  **0.011 (0.002)**** |
| DM | Logical memory 1  Logical memory 2  Faces 1  Faces 2 | **-0.139**  **-0.134**  **-0.120**  -0.095 | **0.019 (0.000)*****  **0.018 (0.000)*****  **0.014 (0.001 )****  0.009 (0.010)* | **-0.155**  **-0.161**  **-0.122**  **-0.101** | **0.024 (0.000)*****  **0.026 (0.000)*****  **0.015 (0.001)****  **0.010 (0.006)**** | **-0.140**  **-0.149**  -0.093  -0.063 | **0.020 (0.000)*****  **0.022 (0.000)*****  0.009 (0.012)*  0.004 (0.087) |
| WM | SWM (errors)  SWM (strategy)  Letter-number sequencing | 0.060  0.036  -0.066 | 0.004 (0.077)  0.001 (0.304)  0.004 (0.030)* | **0.100**  0.058  **-0.107** | **0.010 (0.003)****  0.003 (0.110)  **0.011 (0.000)***** | 0.081  0.025  **-0.091** | 0.006 (0.017)*  0.001 (0.481)  **0.008 (0.003)**** |
| Attention | SART reaction time  CPT d’Prime 2 digit  CPT d’Prime 3 digit  CPT d’Prime 4 digit | 0.093  -0.067  -0.024  0.014 | 0.009 (0.016)*  0.004 (0.134)  0.001 (0.590)  0.000 (0.760) | **0.113**  -0.012  0.005  0.026 | **0.013 (0.003)****  0.000 (0.787)  0.000 (0.920)  0.001 (0.581) | 0.082  0.013  0.017  0.035 | 0.007 (0.033)*  0.000 (0.763)  0.000 (0.709)  0.001 (0.455) |
| Social Cognition | Eyes  Hint  EB  PB | 0.025  -0.066  0.022  0.014 | 0.001 (0.530)  0.004 (0.127)  0.000 (0.615)  0.000 (0.744) | -0.017  **-0.121**  0.032  -0.024 | 0.000 (0.662)  **0.015 (0.005)****  0.001 (0.468)  0.001 (0.596) | -0.016  **-0.116**  0.013  -0.022 | 0.000 (0.678)  **0.014 (0.007)****  0.000 (0.764)  0.000 (0.621) |

**p*<0.05; ***p*<0.01; ****p*<0.0001. Results in bold indicate significance after correction for multiple testing across 5 cognitive domains.

*MIR137*, microRNA 137; IQ, intelligence quotient; WTAR, Wechsler Test of Adult Reading; DM, declarative memory; WM, working memory; SART, Sustained Attention to Response Task; CPT, Continuous Performance Task. MiR-137 polygene scores were thresholded at *p*=10^-5^, *p*=0.05 and *p*=0.5.

**Supplementary Table 5:** Clusters, including individual peaks, showing significantly increased activity with increasing *MIR137* pathway risk score (*p*=10^-5^ level) during increasing spatial working memory load (3 dots versus 1 dot), corrected for multiple comparisons at the cluster-level.

| **Cluster** | **Extent (voxels)** | **p value** | **Cluster peak** | **t-value** | **Z-value** | **Peak coordinates (MNI)** |
| --- | --- | --- | --- | --- | --- | --- |
| 1 | 175 | 0.011 | Right inferior occipital gyrus | 4.80 | 4.49 | 48 -76 -2 |
|  |  |  | Not found on any probability map | 4.73 | 4.43 | 36 -58 1 |
|  |  |  | Right middle temporal gyrus | 4.27 | 4.05 | 54 -67 1 |
| 2 | 121 | 0.038 | Not found on any probability map | 4.19 | 3.97 | 3 -34 16 |
|  |  |  | Not found on any probability map | 3.98 | 3.79 | -9 -40 19 |
|  |  |  | Not found on any probability map | 3.56 | 3.42 | -15 -43 28 |

**Supplementary Table 6:** Clusters, including individual peaks, showing significantly increased activity during increasing spatial working memory load (3 dots versus 1 dot), corrected for multiple comparisons at the cluster-level (N = 83)

| Cluster | Extent (voxels) | p value | Cluster peak | t-value | Z-value | Peak coordinates (MNI) |
| --- | --- | --- | --- | --- | --- | --- |
| 1 | 2154 | <0.001 | Superior Parietal Lobule | 13.06 | >8 | 21 -67 55 |
|  |  |  | Inferior Parietal Lobule | 11.40 | >8 | 42 -40 49 |
|  |  |  | Middle Occipital Gyrus | 11.10 | >8 | 30 -73 28 |
| 2 | 1188 | <0.001 | Superior Parietal Lobule | 9.90 | >8 | -21 -64 58 |
|  |  |  | Middle Occipital Gyrus | 7.84 | 6.76 | -27 -76 31 |
|  |  |  | Inferior Parietal Lobule | 7.23 | 6.34 | -36 -43 46 |
| 3 | 295 | 0.001 | Precentral Gyrus | 8.84 | 7.38 | 27 -4 49 |
| 4 | 149 | 0.022 | Superior Frontal Gyrus | 6.15 | 5.56 | -24 -7 52 |
| 5 | 128 | 0.034 | Superior Medial Gyrus | 5.28 | 4.89 | 6 26 43 |
| 6 | 115 | 0.046 | Inferior Frontal Gyrus (p. Opercularis) | 5.18 | 4.80 | 51 11 28 |
| 7 | 113 | 0.049 | Inferior Temporal Gyrus | 4.91 | 4.58 | -45 -61 -8 |

**References**

Higgins, J. P., & Green, S. (2008). *Cochrane handbook for systematic reviews of interventions* (Vol. 5): Wiley Online Library.
