# Supplementary figures and images for: MiR-137-derived polygenic risk: effects on cognitive performance in patients with schizophrenia and controls
Source: Transl Psychiatry. 2017 Jan 24;7(1):e1012–. doi: 10.1038/tp.2016.286 (PMC5545742; doi:10.1038/tp.2016.286)

**A) No Delay**

Match

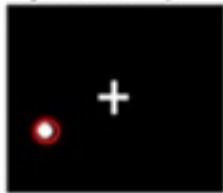

No Match

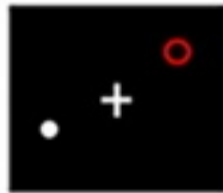

**B) 1-dot**

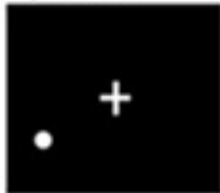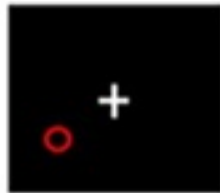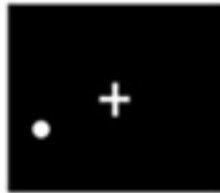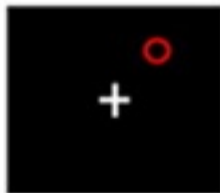

time

**C) 3-dot**

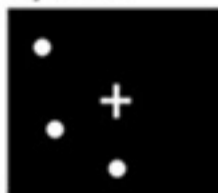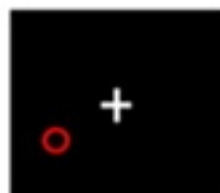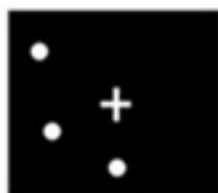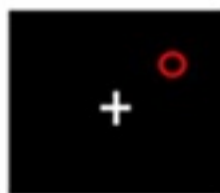

time

Supplement: Supplementary Figure 1 [file tp2016286x2.pdf]
