# Supplementary material for: MiR-137-derived polygenic risk: effects on cognitive performance in patients with schizophrenia and controls
Source: Transl Psychiatry. 2017 Jan 24;7(1):e1012–. doi: 10.1038/tp.2016.286 (PMC5545742; doi:10.1038/tp.2016.286)
Supplement: Supplementary Table 7 [file tp2016286x3.pdf]

chr1	1317275	1362693	MRPL20
chr1	2095898	2164159	C1ORF86
chr1	9769078	9904584	CLSTN1
chr1	9969775	10022826	LZIC
chr1	19177923	19249293	ALDH4A1
chr1	20419142	20466059	PLA2G2D
chr1	20805940	20854674	MUL1
chr1	22870003	22950087	EPHA8
chr1	23611180	23690857	HNRNPR
chr1	32352021	32423988	PTP4A2
chr1	33096748	33171812	RBBP4
chr1	33125506	33188361	SYNC
chr1	36601565	36666451	MAP7D1
chr1	40954432	41002214	EXO5
chr1	41306727	41348018	CITED4
chr1	41472870	41727815	SCMH1
chr1	41807602	41869263	FOXO6
chr1	44392477	44453694	IPO13
chr1	46749284	46802449	UQCRH
chr1	47080710	47154099	ATPAF1
chr1	49173194	49262641	BEND5
chr1	50886934	51445936	FAF1
chr1	52850218	52903992	PRPF38A
chr1	54391998	54453841	LRRC42
chr1	63229776	63350941	ATG4C
chr1	64038946	64145916	PGM1
chr1	65278905	65452187	JAK1
chr1	68874506	68935642	RPE65
chr1	82145454	82478422	ADGRL2
chr1	84944005	84992262	GNG5
chr1	92475532	92549093	EPHX4
chr1	94332589	94395012	GCLM
chr1	95428278	95558507	ALG14
chr1	97523299	98406615	DPYD
chr1	100711713	100778325	RTCA
chr1	109814986	109869663	MYBPHL
chr1	109921652	109989108	PSMA5
chr1	114284453	114375098	RSBN1
chr1	117097019	117230377	IGSF3
chr1	145189112	145306270	NOTCH2NL
chr1	150989028	151040076	BNIPL
chr1	151984981	152029511	S100A11
chr1	153934092	153978853	RAB13
chr1	155003747	155055252	ADAM15
chr1	155087819	155131334	SLC50A1
chr1	155138299	155182706	MUC1
chr1	155196995	155245274	FAM189B
chr1	155258538	155310457	FDPS
chr1	155609232	155678823	YY1AP1
chr1	155958838	156010758	SSR2
chr1	155985084	156043616	UBQLN4
chr1	160165504	160252350	DCAF8
chr1	160987421	161028774	TSTD1
chr1	161121099	161167758	B4GALT3

chr1	161491550	161540413	FCGR3A
chr1	161699557	161746954	DUSP12
chr1	165673527	165758159	TMCO1
chr1	169081767	169357201	NME7
chr1	171649295	171731379	VAMP4
chr1	179831176	179909212	TOR1AIP1
chr1	182595791	182662067	RGS8
chr1	192961495	193049237	UCHL5
chr1	201414606	201458299	PHLDA3
chr1	202827409	202878385	RABIF
chr1	203679704	203720979	LINC00260
chr1	206496199	206657783	SRGAP2
chr1	206744973	206805904	EIF2D
chr1	211479956	211568286	TRAF5
chr1	212718675	212814119	ATF3
chr1	212879494	212985139	NSL1
chr1	222771443	222861354	MIA3
chr1	224281788	224369749	FBXO28
chr1	225977775	226053262	EPHX1
chr1	226230407	226279703	H3F3A
chr1	229386808	229461640	RAB4A
chr1	231021986	231134618	TTC13
chr1	233099881	233451459	PCNXL2
chr1	236692304	236787841	HEATR1
chr1	242138791	242182385	MAP1LC3C
chr1	246683862	246749565	TFB2M
chr1	249124202	249173315	ZNF692
chr2	259560	308308	FAM150B
chr2	18715988	18761959	RDH14
chr2	24787345	25013570	NCOA1
chr2	25942252	26121312	ASXL2
chr2	26765480	26822395	C2orf70
chr2	27173238	27270087	MAPRE3
chr2	27567218	27613324	EIF2B4
chr2	27584065	27652550	PPM1G
chr2	27595489	27636443	FTH1P3
chr2	28954613	29045806	PPP1CB
chr2	29052687	29113175	TRMT61B
chr2	29184163	29295096	FAM179A
chr2	30434396	30502899	LBH
chr2	38502028	38624432	ATL2
chr2	39872637	39965104	TMEM178A
chr2	42255160	42305668	PKDCC
chr2	44375941	44481742	PPM1B
chr2	46718985	46767096	ATP6V1E2
chr2	48647907	48762531	PPP1R21
chr2	53877116	54034146	ASB3
chr2	62403261	62471866	B3GNT2
chr2	64099666	64266214	VPS54
chr2	68674690	68827294	APLF
chr2	74679084	74719942	MRPL53
chr2	85561842	85638875	ELMOD3
chr2	86406555	86460477	MRPL35
chr2	96048447	96098879	FAHD2A

chr2	97729322	97780582	FAHD2B
chr2	99041320	99227496	INPP4A
chr2	100988321	101054130	CHST10
chr2	106978569	107027851	PLGLA
chr2	110530334	111356309	RGPD6
chr2	110685266	111201393	LOC440895
chr2	110949105	111000517	LINC00116
chr2	112953438	113032664	ZC3H8
chr2	131654223	131824826	ARHGEF4
chr2	136851918	136895725	CXCR4
chr2	149874980	150091772	LYPD6B
chr2	175592322	175649200	CHRNA1
chr2	176020985	176066490	ATP5G3
chr2	190591385	190647924	OSGEPL1
chr2	198331307	198384998	HSPD1
chr2	198360294	198438423	MOB4
chr2	198571602	198671036	BOLL
chr2	201960876	202057411	CFLAR
chr2	203725322	203796949	WDR12
chr2	206527223	206682857	NRP2
chr2	209080950	209139867	IDH1
chr2	217516827	217580272	IGFBP5
chr2	220054487	220103712	ABCB6
chr2	220064101	220114410	ATG9A
chr2	220090191	220135059	STK16
chr2	220279699	220378354	SPEG
chr2	220383668	220428487	CHPF
chr2	220472291	220526702	SLC4A3
chr2	227576032	227683506	IRS1
chr2	230879689	230953715	SLC16A14
chr2	233392679	233435300	TIGD1
chr2	234243152	234400743	DGKD
chr2	239120326	239162985	LOC643387
chr2	241479470	241523431	DUSP28
chr2	242234601	242313441	SEPT2
chr3	4382828	4528966	SUMF1
chr3	9671116	9764078	MTMR14
chr3	12174567	12220851	TIMP4
chr3	15431266	15489054	METTL6
chr3	15471639	15583258	COLQ
chr3	20182084	20247724	SGOL1
chr3	32003265	32053228	ZNF860
chr3	32413162	32516333	CMTM7
chr3	33171536	33280707	SUSD5
chr3	42112745	42287382	TRAK1
chr3	42510790	42599065	VIPR1
chr3	43387817	43683560	ANO10
chr3	43712374	43784217	ABHD5
chr3	45103765	45207914	CDCP1
chr3	47402471	47474931	PTPN23
chr3	47517129	47575199	ELP6
chr3	48705435	48774711	IP6K2
chr3	49037907	49080926	NDUFAF3
chr3	49741727	49843973	IP6K1

chr3	50209042	50255129	GNAT1
chr3	50342339	50385669	TUSC2
chr3	51982525	52028646	ABHD14B
chr3	52550620	52594586	SMIM4
chr3	58203258	58300461	ABHD6
chr3	58393356	58439579	PDHB
chr3	58457822	58508087	KCTD6
chr3	58529838	58633337	FAM107A
chr3	73411580	73694072	PDZRN3
chr3	109108836	109234014	LINC01205
chr3	119127806	119202529	TMEM39A
chr3	121468609	121573926	IQCB1
chr3	122493900	122619986	DIRC2
chr3	124429212	124488119	UMPS
chr3	124924512	125114198	ZNF148
chr3	125705199	125840391	SLC41A3
chr3	127387904	127562093	MGLL
chr3	128786411	128860993	RAB43
chr3	128866657	128922810	CNBP
chr3	131232403	131778450	CPNE4
chr3	133272433	133329118	CDV3
chr3	134054186	134114259	AMOTL2
chr3	137886089	138037228	ARMC8
chr3	139042860	139095887	MRPS22
chr3	143670639	143731210	C3ORF58
chr3	151024095	151067337	P2RY13
chr3	165470691	165575253	BCHE
chr3	170760291	171198197	TNIK
chr3	180610233	180720539	FXR1
chr3	181409711	181452223	SOX2
chr3	183940116	183987313	ALG3
chr3	189654516	189860226	P3H2
chr3	190211839	190394986	IL1RAP
chr3	194055975	194110472	LRRC15
chr3	196261058	196315413	WDR53
chr3	196642272	196689464	NCBP2
chr4	186388	269773	ZNF876P
chr4	646224	688127	ATP5I
chr4	651710	695817	MYL5
chr4	985609	1040686	FGFRL1
chr4	1045265	1127352	RNF212
chr4	1263638	1353936	MAEA
chr4	2023719	2065697	C4ORF48
chr4	6621817	6664470	MRFAP1
chr4	17558926	17629590	LAP3
chr4	20682035	20749980	PACRGL
chr4	39026450	39147853	KLHL5
chr4	48813059	48883834	OCIAD1
chr4	76922268	76964689	CXCL10
chr4	83530689	83740010	SCD5
chr4	83719813	83832433	SEC31A
chr4	88908798	89018931	PKD2
chr4	100717980	100811346	DAPP1
chr4	122725483	122811652	BBS7

chr4	128962420	129152298	LARP1B
chr4	140167316	140221492	MGARP
chr4	145547147	145679881	HHIP
chr4	150979425	151198608	DCLK2
chr4	152004978	152045043	SNORD73A
chr4	157977276	158113242	GLRB
chr4	169398216	169869608	PALLD
chr4	184406202	184453581	ING2
chr4	189040597	189088649	TRIML1
chr5	7839271	7889150	FASTKD3
chr5	31512372	31575165	C5ORF22
chr5	32207112	32333114	MTMR12
chr5	32334455	32464844	ZFR
chr5	34895819	34945787	BRIX1
chr5	40811429	40855387	RPL37
chr5	43019181	43060447	ANXA2R
chr5	64865506	64940187	TRIM23
chr5	64900557	64981954	TRAPPC13
chr5	71495235	71636084	MRPS27
chr5	72774249	72821448	BTF3
chr5	79902044	79970800	DHFR
chr5	79930466	80192634	MSH3
chr5	80577401	80628965	ZCCHC9
chr5	95977740	96130385	CAST
chr5	96191643	96275406	ERAP2
chr5	102181526	102386808	PAM
chr5	110407869	110486200	WDR36
chr5	125916606	125982944	PHAX
chr5	132367661	132460709	HSPA4
chr5	137455458	137534358	BRD8
chr5	138069084	138290723	CTNNA1
chr5	138262409	138554065	SIL1
chr5	138589440	138687366	MATR3
chr5	139534652	139643374	CYSTM1
chr5	139999011	140044993	TMCO6
chr5	140004947	140047370	NDUFA2
chr5	140835568	140912544	PCDHGC3
chr5	145472588	145582294	LARS
chr5	146750370	146909619	DPYSL3
chr5	147238273	147281756	SCGB3A2
chr5	150020402	150078930	MYOZ3
chr5	151020656	151086615	SPARC
chr5	154178051	154250213	FAXDC2
chr5	167699064	167919308	WWC1
chr5	171268555	171453877	FBXW11
chr5	172241222	172399688	ERGIC1
chr5	176712500	176759292	MXD3
chr5	176764843	176819599	RGS14
chr5	176863613	176920694	DBN1
chr5	176890394	176944606	PDLIM7
chr5	179204597	179253952	MGAT4B
chr5	180197540	180257137	MGAT1
chr6	2980049	3040110	NQO2
chr6	7261375	7333547	SSR1

chr6	10675187	10729970	PAK1IP1
chr6	13595558	13641127	NOL7
chr6	26614610	26679980	ZNF322
chr6	28173028	28221264	ZSCAN9
chr6	31058999	31100332	C6ORF15
chr6	31810969	31866823	SLC44A4
chr6	32132509	32177963	PBX2
chr6	33224916	33266602	B3GALT4
chr6	33247471	33302164	TAPBP
chr6	33266334	33310793	DAXX
chr6	33644537	33699528	UQCC2
chr6	34535056	34684627	C6ORF106
chr6	42911610	42966981	PEX6
chr6	53639777	53808919	LRRC1
chr6	74205472	74250755	EEF1A1
chr6	86297501	86373043	SYNCRIP
chr6	87627023	87746397	HTR1E
chr6	91203291	91317020	MAP3K7
chr6	108168959	108299482	SEC63
chr6	110481623	110573422	CDC40
chr6	110911180	111157088	CDK19
chr6	116872529	116934764	RWDD1
chr6	118208688	118658839	SLC35F1
chr6	129184285	129857710	LAMA2
chr6	132249316	132292518	CTGF
chr6	134470383	134659196	SGK1
chr6	137498620	137560567	IFNGR1
chr6	149048270	149418126	UST
chr6	149896010	149989940	KATNA1
chr6	158713691	158952856	TULP4
chr6	159037506	159085818	DYNLT1
chr6	160128029	160197352	WTAP
chr6	166758407	166816501	MPC1
chr6	170864659	170913780	PDCD2
chr7	1835427	2292583	MAD1L1
chr7	4993615	5057800	RNF216P1
chr7	5326422	5483177	TNRC18
chr7	5546778	5590232	ACTB
chr7	5918340	5985603	CCZ1
chr7	6635526	6683921	ZNF853
chr7	6656952	6717910	ZNF316
chr7	16773350	16844161	TSPAN13
chr7	20801893	20846508	SP8
chr7	38197807	38290272	STARD3NL
chr7	40152341	40194251	MPLKIP
chr7	40154574	40920366	SUGCT
chr7	43895492	43986010	URGCP
chr7	45119698	45171346	TBRG4
chr7	55518300	55660200	VOPP1
chr7	64478731	64555091	CCT6P3
chr7	66073867	66128216	KCTD7
chr7	66127077	66296448	RABGEF1
chr7	72398831	72445302	NSUN5P2
chr7	73130424	73173197	ABHD11

chr7	73683804	73840273	CLIP2
chr7	73848119	74036920	GTF2IRD1
chr7	75488316	75538244	RHBDD2
chr7	75911874	75953614	HSPB1
chr7	86805477	86869031	TMEM243
chr7	89955978	90040769	GTPBP10
chr7	95014173	95084384	PON2
chr7	97461428	97521854	ASNS
chr7	99035783	99083824	ATP5J2
chr7	99885324	99939819	SPDYE3
chr7	99935625	99985454	PILRB
chr7	100151633	100203811	LRCH4
chr7	102257471	102303238	UPK3BL
chr7	102932920	103005320	DNAJC2
chr7	116572500	116614388	ST7-AS1
chr7	120968904	121056422	FAM3C
chr7	127617561	127660130	SND1-IT1
chr7	128675276	128717293	TPI1P2
chr7	129054273	129148239	STRIP2
chr7	138125078	138290332	TRIM24
chr7	138708265	138814465	ZC3HAV1
chr7	140013551	140118350	SLC37A3
chr7	143058359	143108206	ZYX
chr7	150056405	150115719	ZNF775
chr7	150735298	150793614	SLC4A2
chr7	150753707	150797970	FASTK
chr7	154775142	154817413	PAXIP1-AS1
chr7	156911654	157082066	UBE3C
chr7	157109709	157230133	DNAJB6
chr8	21944382	21986932	NUDT18
chr8	27471576	27554286	SCARA3
chr8	28727910	28930242	HMBOX1
chr8	30870777	31051277	WRN
chr8	37696464	37777015	RAB11FIP1
chr8	37943010	38017598	ASH2L
chr8	38069008	38140287	DDHD2
chr8	42975591	43077970	HGSNAT
chr8	67935314	67994562	COPS5
chr8	74186836	74257520	RDH10
chr8	82549150	82618589	IMPA1
chr8	85999322	86078314	LRRCC1
chr8	90749974	90823292	RIPK2
chr8	96017213	96090944	NDUFAF6
chr8	98861310	99068946	MATN2
chr8	100005493	100909814	VPS13B
chr8	117838172	117907105	RAD21
chr8	120408551	120456678	NOV
chr8	124312090	124428705	ATAD2
chr8	130831838	131048897	FAM49B
chr8	132896355	133045886	EFR3A
chr8	145731602	145774458	C8orf82
chr9	3804127	4320035	GLIS3
chr9	6393150	6527051	UHRF2
chr9	19095758	19147604	PLIN2

chr9	20986364	21051635	HACD4
chr9	26883367	26967468	PLAA
chr9	27089138	27250176	TEK
chr9	27526543	27593864	C9ORF72
chr9	38372660	38418662	ALDH1B1
chr9	70950079	70992782	PGM5-AS1
chr9	71716179	71890124	TJP2
chr9	78993514	79034954	RPSAP9
chr9	96188781	96235874	FAM120AOS
chr9	99055718	99165992	SLC35D2
chr9	99771958	99821925	CTSV
chr9	112117973	112280593	PTPN3
chr9	114102972	114267025	KIAA0368
chr9	116017913	116075185	PRPF4
chr9	117761853	117900536	TNC
chr9	123131146	123362448	CDK5RAP2
chr9	123597928	123677174	PHF19
chr9	130608758	130660022	AK1
chr9	130853449	130901013	SLC25A25-AS1
chr9	130902538	130946207	C9ORF16
chr9	131051395	131104697	TRUB2
chr9	131917830	131960540	IER5L
chr9	132914856	133019583	NCS1
chr9	133434959	133533739	FUBP3
chr9	134145080	134204649	PPAPDC3
chr9	134715496	134975274	MED27
chr9	139599045	139642636	SNHG7
chr9	140063053	140104822	SSNA1
chr9	140099086	140140763	CYSRT1
chr9	140297846	140348858	NOXA1
chr9	140426308	140467007	MRPL41
chr10	1014348	1083708	GTPBP4
chr10	5070957	5169878	AKR1C3
chr10	5883688	5951860	ANKRD16
chr10	7810092	7869762	ATP5C1
chr10	13299795	13362130	PHYH
chr10	23708197	23751310	OTUD1
chr10	27379039	27463349	YME1L1
chr10	30961202	31025942	SVILP1
chr10	33169245	33267293	ITGB1
chr10	33446418	33643833	NRP1
chr10	35907176	35950362	FZD8
chr10	38625307	38687433	HSD17B7P2
chr10	38697073	38761081	LINC00999
chr10	45451708	45494330	C10ORF10
chr10	46717612	48972629	BMS1P5
chr10	61390521	61489649	SLC16A9
chr10	70080863	70187051	RUFY2
chr10	71942585	72013190	PPA1
chr10	74431888	74667452	MCU
chr10	74874281	74947853	ECD
chr10	75737871	75899914	VCL
chr10	80808791	81096285	ZMIZ1
chr10	88789958	88874776	GLUD1

chr10	93646344	93689258	FGFBP3
chr10	95046185	95262074	MYOF
chr10	97403152	97473900	TCTN3
chr10	102113332	102168111	OLMALINC
chr10	102717578	102767272	MRPL43
chr10	104142373	104199691	PSD
chr10	111745725	111915323	ADD3
chr10	112611552	112679764	PDCD4
chr10	113889621	113963537	GPAM
chr10	115919028	116012063	TDRD1
chr10	116561502	116679586	FAM160B1
chr10	119744426	119826114	RAB11FIP2
chr10	121312977	121376541	TIAL1
chr10	124719555	124769907	PSTK
chr10	126065871	126127545	OAT
chr10	129685324	129904164	PTPRE
chr10	133727954	133793338	PPP2R2D
chr10	135118927	135170475	CALY
chr11	474511	527283	RNH1
chr11	1948501	1997839	MRPL23
chr11	2401717	2445108	TSSC4
chr11	6622554	6697080	DCHS1
chr11	7988866	8037719	EIF3F
chr11	27495964	27548326	LIN7C
chr11	27656441	27763605	BDNF
chr11	32894723	33021814	QSER1
chr11	33086129	33203037	CSTF3
chr11	35619734	35662421	FJX1
chr11	43682142	43898169	HSD17B12
chr11	46745083	46887859	CKAP5
chr11	47566981	47615013	PTPMT1
chr11	61046918	61120684	DDB1
chr11	62360212	62402592	ROM1
chr11	62410288	62459241	LBHD1
chr11	62455065	62496678	GNG3
chr11	62599459	62643360	SNHG1
chr11	63722078	63764015	COX8A
chr11	63733324	63785892	OTUB1
chr11	63977752	64021753	DNAJC4
chr11	64106624	64159687	RPS6KA4
chr11	64512075	64566316	SF1
chr11	65323508	65380116	EHBP1L1
chr11	65581409	65641172	SNX32
chr11	66386087	66455858	RBM4
chr11	67036761	67089955	ANKRD13D
chr11	67902329	68001239	SUV420H1
chr11	71619767	71728643	RNF121
chr11	74951165	75082875	ARRB1
chr11	75506211	75875282	UVRAG
chr11	76041000	76112009	PRKRIR
chr11	85385264	85542202	SYTL2
chr11	89913596	89976532	CHORDC1
chr11	93444144	93484265	SNORA32
chr11	102168180	102230135	BIRC3

chr11	107972257	108038891	ACAT1
chr11	111632918	111762305	ALG9
chr11	114290107	114341000	REXO2
chr11	118947212	118992785	DPAGT1
chr11	120087348	120210653	POU2F3
chr11	120187617	120380645	ARHGEF12
chr11	122506397	122705187	UBASH3B
chr11	122733235	122850430	C11ORF63
chr11	125295640	125386206	FEZ1
chr12	4362901	4434522	CCND2
chr12	6623570	6667541	GAPDH
chr12	6813149	6861041	COPS7A
chr12	7945107	8063792	SLC2A14
chr12	8266364	8307448	POU5F1P3
chr12	8778538	8835484	MFAP5
chr12	10831675	10895953	YBX3
chr12	12946279	13002915	DDX47
chr12	22179109	22238606	CMAS
chr12	32812133	32918584	DNM1L
chr12	45589769	45854187	ANO6
chr12	49026994	49096035	KANSL2
chr12	49188214	49242726	CACNB3
chr12	49295741	49339330	FKBP11
chr12	50485763	50534240	COX14
chr12	53380041	53455993	EIF4B
chr12	53825885	53894946	PCBP2
chr12	53854275	53913444	MAP3K12
chr12	56478102	56527694	PA2G4
chr12	59245936	59334319	LRIG3
chr12	64778152	64862463	XPOT
chr12	65543350	65662141	LEMD3
chr12	66496848	66544533	LLPH
chr12	72059877	72117839	TMEM19
chr12	77395025	77479360	E2F7
chr12	98967402	99015778	SLC25A3
chr12	100410862	100556642	UHRF1BP1L
chr12	104362764	104463915	GLT8D2
chr12	104438235	104520304	HCFC2
chr12	110268747	110338293	GLTP
chr12	110417234	110497235	ANKRD13A
chr12	110886231	110948192	FAM216A
chr12	112444492	112566635	NAA25
chr12	120759132	120826983	MSI1
chr12	120864240	120921556	GATC
chr12	121180312	121362155	SPPL3
chr12	122936145	123005620	ZCCHC8
chr12	123217367	123275611	DENR
chr12	123935908	124038265	RILPL1
chr12	124437761	124519986	ZNF664
chr12	130627003	130670285	FZD10
chr12	132414464	132585011	EP400
chr13	20377623	20457776	ZMYM5
chr13	35496423	36266874	NBEA
chr13	39897028	40197356	LHFP

chr13	41486054	41613508	ELF1
chr13	42011541	42065013	RGCC
chr13	45493383	45583613	NUFIP1
chr13	48965181	49038840	LPAR6
chr13	49862785	50038262	CAB39L
chr13	52566522	52623780	ALG11
chr13	71992097	72461330	DACH1
chr13	78449615	78569664	EDNRB
chr13	79168420	79253314	RNF219
chr13	101162417	101261046	GGACT
chr13	114090133	114165023	DCUN1D2
chr14	20791772	20846063	PARP2
chr14	21538204	21592863	ZNF219
chr14	24563905	24614451	DCAF11
chr14	31071459	31225033	SCFD1
chr14	34882143	34951468	SPTSSA
chr14	35159587	35204029	CFL2
chr14	36275596	36361169	BRMS1L
chr14	39563487	39626177	GEMIN2
chr14	50067488	50110199	MGAT2
chr14	50071891	50121948	DNAAF2
chr14	50979799	51119784	ATL1
chr14	52096235	52138462	FRMD6-AS1
chr14	53153895	53214716	PSMC6
chr14	53176882	53261705	STYX
chr14	55498361	55556912	MAPK1IP1L
chr14	57715605	57776797	AP5M1
chr14	58450807	58638847	C14ORF37
chr14	58691522	58758727	PSMA3
chr14	60591499	60652211	DHRS7
chr14	63651101	63780230	RHOJ
chr14	65151192	65231060	PLEKHG3
chr14	73937638	73980105	C14ORF169
chr14	77233585	77299283	ANGEL1
chr14	90986931	91302761	TTC7B
chr14	92568297	92650543	CPSF2
chr14	93383258	93602263	ITPK1
chr14	93649236	93693459	C14ORF142
chr14	94527638	94589060	IFI27L1
chr14	94574117	94615957	IFI27L2
chr14	96132753	96178980	TCL1B
chr14	96947984	96988663	LOC730202
chr14	100130754	100213638	CYP46A1
chr14	100239744	100428395	EML1
chr14	103975508	104023410	TRMT61A
chr14	105246932	105291048	ZBTB42
chr14	105432615	105481855	C14ORF79
chr15	22813394	22893891	TUBGCP5
chr15	23868695	23912993	MAGEL2
chr15	34356223	34414053	EMC7
chr15	34497197	34542366	EMC4
chr15	40206324	40347797	EIF2AK4
chr15	42821010	42882190	HAUS2
chr15	49010134	49123343	CEP152

chr15	63776709	63906839	USP3
chr15	64344757	64406207	FAM96A
chr15	65235361	65302284	SPG21
chr15	66974673	67094337	SMAD6
chr15	72513521	72583628	PARP6
chr15	74813547	74910472	ARID3B
chr15	74880712	74942542	CLK3
chr15	75641719	75768124	SIN3A
chr15	75739461	75891632	PTPN9
chr15	78779905	78849715	HYKK
chr15	90273097	90314540	MESP1
chr15	91240557	91378692	BLM
chr15	91453409	91517323	UNC45A
chr15	92376937	92735665	SLCO3A1
chr15	101791113	101837725	VIMP
chr15	102481015	102536808	WASH3P
chr16	107017	155850	MPG
chr16	115803	208697	NPRL3
chr16	202845	243709	HBA2
chr16	426754	470754	NME4
chr16	751141	792590	FAM173A
chr16	2008917	2051550	NOXO1
chr16	2069815	2117867	NTHL1
chr16	3313486	3361459	ZNF263
chr16	3431189	3479364	ZNF174
chr16	4384542	4486962	CORO7
chr16	4638883	4684927	UBALD1
chr16	8869036	8911505	TMEM186
chr16	11621577	11701322	LITAF
chr16	19707777	19889789	IQCK
chr16	21340683	21417254	SNX29P1
chr16	22337256	22405938	CDR2
chr16	29444913	29486285	BOLA2
chr16	29953350	30004373	TMEM219
chr16	30689024	30729810	LOC730183
chr16	30940404	30986259	ORAI3
chr16	46703557	46752306	ORC6
chr16	56498258	56574008	BBS2
chr16	58477548	58567523	NDRG4
chr16	66521905	66604315	TK2
chr16	67403709	67447438	TPPP3
chr16	67886925	67938417	EDC4
chr16	67957376	68022597	SLC12A4
chr16	69353414	69397013	NIP7
chr16	69990201	70119851	PDXDC2P
chr16	70701341	70855061	VAC14
chr16	72107614	72166811	DHX38
chr16	78113309	79266564	WWOX
chr16	89554795	89644176	SPG7
chr16	89607064	89653237	RPL13
chr16	89733075	89782772	CDK10
chr16	89753540	89807394	VPS9D1
chr16	90051272	90106539	DBNDD1
chr17	1304646	1379561	CRK

chr17	1347479	1416001	MYO1C
chr17	4828944	4872381	PFN1
chr17	6461644	6564247	KIAA0753
chr17	6895735	6937852	RNASEK
chr17	7462804	7505429	CD68
chr17	7474547	7538215	FXR2
chr17	7529944	7581089	ATP1B2
chr17	8223187	8269363	ODF4
chr17	16264106	16306059	UBB
chr17	18833988	18944004	SLC5A10
chr17	26664603	26710705	TMEM199
chr17	27035831	27089784	NEK8
chr17	28883482	28984484	LRRC37BP1
chr17	29206000	29253286	TEFM
chr17	30449472	30572790	RHOT1
chr17	34502268	34544157	CCL3L1
chr17	34822470	34875154	ZNHIT3
chr17	36986320	37030053	RPL23
chr17	37865408	37906788	MIEN1
chr17	40741357	40787256	TUBG1
chr17	41146621	41194459	VAT1
chr17	41157257	41204058	RND2
chr17	42907654	42996993	EFTUD2
chr17	45311207	45410077	ITGB3
chr17	54849273	54931256	C17ORF67
chr17	56402535	56449599	SUPT4H1
chr17	56577610	56638179	SEPT4
chr17	57764862	57937952	VMP1
chr17	62474373	62522484	DDX5
chr17	71141159	71188062	SSTR2
chr17	72179794	72226019	RPL38
chr17	72407666	72463568	GPRC5C
chr17	73643195	73724142	SAP30BP
chr17	74688913	74742881	JMJD6
chr17	74702911	74749963	METTL23
chr17	76829058	76941472	TIMP2
chr17	78055354	78113679	GAA
chr17	78214659	78392581	RNF213
chr17	78420632	78470404	NPTX1
chr17	79198610	79289139	SLC38A10
chr17	79628223	79670954	ARL16
chr18	3391924	3478406	TGIF1
chr18	3583734	3630087	DLGAP1-AS2
chr18	9688227	9882553	RAB31
chr18	12287667	12349824	TUBB6
chr18	12765476	12904334	PTPN2
chr18	32800993	32858397	ZNF397
chr18	33668493	33729357	SLC39A6
chr18	43644109	43704199	ATP5A1
chr18	44036934	44256996	LOXHD1
chr18	77846914	77918228	ADNP2
chr19	285574	364791	MIER2
chr19	1363882	1415588	NDUFS7
chr19	1461426	1510449	PCSK4

chr19	1795244	1868452	REXO1
chr19	2308628	2375100	SPPL2B
chr19	3524196	3577582	MFSD12
chr19	3749088	3792219	RAX2
chr19	5810636	5859742	FUT6
chr19	6643147	6690599	TNFSF14
chr19	7579037	7646653	PNPLA6
chr19	8003456	8090529	ELAVL1
chr19	10050236	10141147	COL5A3
chr19	10808728	10962586	DNM2
chr19	10962252	11053448	CARM1
chr19	12779729	12827455	FBXW9
chr19	13036627	13084457	RAD23A
chr19	13865256	13909586	C19ORF53
chr19	13886273	13963044	ZSWIM4
chr19	15250443	15331792	NOTCH3
chr19	15919756	15967131	UCA1
chr19	16415650	16458339	KLF2
chr19	16920197	17011164	SIN3B
chr19	17166590	17344104	MYO9B
chr19	17493747	17536457	BST2
chr19	17602431	17652097	PGLS
chr19	17810302	17865324	MAP1S
chr19	18703681	18751849	TMEM59L
chr19	18922743	18999041	UPF1
chr19	19714465	19759039	LPAR2
chr19	20258022	20331299	ZNF486
chr19	30282900	30335215	CCNE1
chr19	33067906	33186102	ANKRD27
chr19	33349903	33482935	CEP89
chr19	35739895	35790718	USF2
chr19	36213427	36256343	U2AF1L4
chr19	36216477	36258056	PSENEN
chr19	36916020	37000804	ZNF566
chr19	37618339	37683643	ZNF585A
chr19	38873774	38919728	FAM98C
chr19	39401347	39443659	MRPS12
chr19	40002172	40043507	EID2B
chr19	40009446	40050838	EID2
chr19	41079071	41155725	LTBP4
chr19	41705103	41787672	AXL
chr19	41911263	41954635	B3GNT8
chr19	42440832	42483528	RABAC1
chr19	42731712	42779316	ERF
chr19	42752688	42819948	CIC
chr19	42781184	42826952	PAFAH1B3
chr19	42871170	42914444	CNFN
chr19	44396775	44459411	ZNF45
chr19	44509493	44557262	ZNF222
chr19	45522297	45594214	CLASRP
chr19	49448565	49490136	FTL
chr19	49451381	49516610	GYS1
chr19	50989253	51034612	JOSD2
chr19	51850351	51892257	CLDND2

chr19	52339055	52411229	ZNF577
chr19	52372487	52428305	ZNF649
chr19	54348837	54399689	MYADM
chr19	54474402	54535920	CACNG6
chr19	55365548	55422786	FCAR
chr19	55870611	55915627	TMEM238
chr19	58316666	58358830	FKBP1AP1
chr19	58924180	58971589	ZNF132
chr20	368708	431610	RBCK1
chr20	1329620	1393816	FKBP1A
chr20	3123262	3174238	LZTS3
chr20	3188062	3239887	SLC4A11
chr20	5075598	5127268	PCNA
chr20	24923579	24993425	APMAP
chr20	25156338	25227360	ENTPD6
chr20	25255378	25391618	ABHD12
chr20	31387698	31458211	MAPRE1
chr20	32057927	32257837	CBFA2T2
chr20	32848070	32919608	AHCY
chr20	33282577	33433433	NCOA6
chr20	33412522	33480661	GGT7
chr20	33442765	33535769	ACSS2
chr20	33496235	33563601	GSS
chr20	34267231	34308902	ROMO1
chr20	34874244	35177040	DLGAP4
chr20	35149886	35198226	MYL9
chr20	43360444	43458979	RIMS4
chr20	44958166	45013097	SLC35C2
chr20	46266149	46435360	SULF2
chr20	49531404	49595060	DPM1
chr20	52804501	52856492	PFDN4
chr20	56864770	56962563	RAB22A
chr20	58491886	58535352	PPP1R3D
chr20	61452365	61513115	TCFL5
chr20	62132132	62173524	PPDPF
chr20	62592430	62684453	PRPF6
chr20	62691470	62751996	OPRL1
chr21	9887188	9988594	TEKT4P2
chr21	34086209	34164169	PAXBP1
chr21	34840361	34884030	DNAJC28
chr21	38103188	38382545	HLCS
chr21	38425570	38595408	TTC3
chr21	45173545	45216256	CSTB
chr21	46687966	46737269	LOC642852
chr21	46914628	46982385	SLC19A1
chr22	17620278	17666335	CECR5-AS1
chr22	18880286	18944066	PRODH
chr22	21251713	21328037	CRKL
chr22	21349441	21402302	P2RX6
chr22	22093946	22241970	MAPK1
chr22	24088020	24130159	CHCHD10
chr22	24293553	24342019	DDT
chr22	25403940	25613415	KIAA1671
chr22	26901713	27006089	TPST2

chr22	29148661	29205289	CCDC117
chr22	29682984	29728778	GAS2L1
chr22	31028037	31083877	DUSP18
chr22	31480762	31523551	SELM
chr22	35633444	35711800	HMGXB4
chr22	37845100	37902478	MFNG
chr22	38282154	38358465	MICALL1
chr22	38844066	38899452	KDELR3
chr22	39416672	39471975	APOBEC3F
chr22	40786284	41052723	MKL1
chr22	42209082	42323312	SREBF2
chr22	42959726	43030968	POLDIP3
chr22	42993845	43065405	CYB5R3
chr22	43172531	43273408	ARFGAP3
chr22	46461876	46529808	MIRLET7BHG
chr22	46643860	46709905	TTC38
chr22	46711297	46773237	TRMU
chr22	50969540	51021328	SYCE3
chr22	50997386	51041428	CHKB
chr22	51041181	51086601	ARSA
chr22	51093069	51191640	SHANK3
